# Imagery-based fear conditioning enhances the late positive potential

**DOI:** 10.3758/s13415-025-01385-y

**Published:** 2026-01-08

**Authors:** Christian Panitz, Erik M. Mueller

**Affiliations:** 1 Department of Psychology, University of Bremen, Hochschulring 18, 28359 Bremen, Germany; 2 Department of Psychology, University of Marburg, Gutenbergstr. 18, 35032 Marburg, Germany

**Keywords:** Mental imagery, Late positive potential, Learning, Threat conditioning, Aversive conditioning, Anxiety

## Abstract

Pairing a conditioned stimulus (CS) repeatedly with aversive imagery as unconditioned stimulus induces conditioned subjective and autonomic fear responses, which can explain fears without prior aversive experiences. To investigate underlying brain dynamics, we assessed the late positive potential, an electrocortical response sensitive to classically conditioned fear, together with skin conductance, cardiac interbeat interval, and fear ratings in 48 participants. Participants underwent either 1) imagery-based fear conditioning, in which aversive CS+ (CS+_av_), neutral CS+ (CS+_neu_), and CS− were paired with cues to imagine an aversive electric shock, a mild vibration, or nothing, respectively, or 2) classical fear conditioning in which CS were paired with physical shocks, physical vibrations, or nothing. Similar to the classical conditioning group, the imagery-based conditioning group reported higher fear for CS+_av_ than for both nonaversive CS and displayed increased late positive potential amplitudes for CS+_av_ compared with CS− and with CS+_neu_. Moreover, the magnitude of these effects did not differ significantly between imagery-based and classical conditioning. Meanwhile, strong cardiac deceleration and increased skin conductance responses to CS+_av_ were uniquely observed for classical conditioning. These results suggest that imagery-based conditioning evokes similar cortical and subjective, but not autonomic, responses as conditioning with physical unconditioned stimuli—advancing our understanding of fear in the absence of aversive real-life experiences.

**Supplementary Information:**

The online version contains supplementary material available at 10.3758/s13415-025-01385-y.

## Introduction

Classical fear conditioning is assumed to play a central role in the acquisition and maintenance of fear and anxiety (Beckers et al., 2023; Duits et al., 2015; Lissek et al., 2005; Mineka & Oehlberg, 2008; Reiss, 1980). At their core, conditioning theories postulate that organisms form associations between previously neutral stimuli (conditioned stimuli [CS]) with inherently aversive stimuli (unconditioned stimuli [US]) that repeatedly follow CS occurrence (Pavlov, 1927; Pearce & Hall, 1980; Rescorla & Wagner, 1972). Over the learning process, CS start to evoke conditioned responses in anticipation of the US. Classical fear conditioning provides a widely accepted and validated theoretical framework to describe and explain experience-based learning of threat cues and defensive responses (Duits et al., 2015; Lissek et al., 2005; Miskovic & Keil, 2012; Wolpe, 1981). Given the theory’s close relation to the behaviorist research tradition, conditioning experiments have almost exclusively paired CS with physical US. Studies on fear acquisition using physical US are well-suited to investigate mechanisms involved in developing fear of dogs after being bitten or barked at, for example, or developing social phobia after repeated embarrassment in front of others. Most of the classical conditioning literature, however, does not provide a straightforward explanation for the many cases of fears and phobias that develop even though individuals cannot report repeated real-life encounters with aversive stimuli (King, 1973).

In order to explain the development and maintenance of fears in the absence of direct experiences, research has explored a potential role of mental imagery of aversive events in conditioning processes (Dadds et al., 1997; King, 1973; Mertens et al., 2020). Given the similarity of psychophysiological responses to physical and imagined stimuli (Cuthbert et al., 2003; Jackson et al., 2006; Ogino et al., 2007; Vrana & Lang, 1990), it has been argued that aversive imagery has the potential to essentially function as an unconditioned stimulus. Conditioned stimuli paired with imagery of a US may evoke similar conditioned responses as when followed by physical US. In support of this hypothesis, previously established associations between the CS and a physical US were stabilized in individuals rehearsing the US after initial learning, and this stabilizing effect of imagery was evident in their conditioned skin conductance responses (Jones & Davey, 1990). In addition to stabilizing effects, more recent evidence suggests that aversive imagery alone appears to be sufficient to create novel CS-US associations, that is, de novo fear conditioning based on imagery rather than any experiences of a physical US (Mueller et al., 2019). Participants reported stronger feelings of unpleasantness, arousal, and fear to CS that had been paired with aversive imagery while also showing conditioned responses in the peripheral autonomic system. Taken together, previous empirical evidence supports the notion that imagery may not only facilitate maintenance of previously acquired CS-US associations, but it even may explain new learning in the absence of physical aversive US. However, this evidence is limited to subjective and peripheral physiological responses. Meanwhile, central neurophysiological markers are needed to better understand the degree to which mechanisms of imagery-based conditioning overlap with those of classical fear conditioning.

One neurophysiological response that may be modulated by imagery-based fear conditioning is the late positive potential (LPP), a sustained positive deflection in the event-related potential (ERP) beginning at around 300 to 400 ms after the onset of arousing and/or motivationally relevant visual stimuli (Cuthbert et al., 2000; Schupp et al., 2000). Recent studies have established increased LPP amplitudes as a reliable response to fear CS (Bacigalupo & Luck, 2018; Ferreira de Sá et al., 2019; Nelson et al., 2015; Panitz et al., 2015, 2018; Sperl et al., 2021; Stolz et al., 2019). Given that the LPP has been hypothesized to reflect increased attentional and emotional engagement (Gupta et al., 2019; Wieser & Keil, 2020), it could provide more direct evidence that CS in imagery-based fear conditioning paradigms actually acquire threat characteristics and trigger central responses of sustained selective attention toward them.

The current study aimed to further investigate the profile of conditioned responses acquired with imagery US and whether they are comparable to responses acquired with physical US. In one of two experimental groups, we used a previously introduced imagery-based fear conditioning paradigm (Mueller et al., 2019) and assessed the LPP in addition to subjective ratings and peripheral measures of autonomic activity, interbeat interval (IBI), and skin conductance responses (SCR). Increased IBIs (i.e., cardiac slowing or “fear bradycardia”) and SCR amplitudes to aversive CS are well-established responses in classical fear conditioning using physical US (Lonsdorf et al., 2017; Mueller et al., 2014; Notterman, 1952; Panitz et al., 2018; Sperl et al., 2016). Meanwhile, there is much less work on autonomic responses in fear conditioning using imagery as US. In a previous study, we found some evidence for cardiac slowing in imagery-based conditioning but none for increased SCRs across two samples (Mueller et al., 2019). Accordingly, we expected relatively increased LPP amplitudes, increased fear ratings, and fear bradycardia to aversively conditioned stimuli within the imagery-based conditioning experimental group. Moreover, while previous studies using imagery-based conditioning have shown response patterns that *qualitatively* resembled those found in other classical conditioning studies, it is not clear whether response magnitudes are comparable *quantitatively.* This is an important question with regard to the phenomenon’s clinical relevance. In principle it may be that the fear responses created by imagery-based fear conditioning are much weaker compared with fear originating from physical experience and therefore negligible from a clinical perspective. Interestingly, previous studies found both smaller (Holland, 1990) or larger (Jones & Davey, 1990) response magnitudes to imagery versus physical stimulation (i.e., unconditioned responses). Meanwhile, there is no direct evidence to whether response magnitudes after imagery-based fear conditioning are smaller, larger, or comparable to classical conditioning. We therefore directly tested potential differences between imagery-based and classical conditioning by including a second experimental group that received physical US with equal learning parameters (i.e., CS material, CS-US interval, number of trials, reinforcement rates). The statistical comparisons of the differential conditioning effects between the two groups were exploratory and nondirected, and Bayesian analyses were used to collect evidence both for differing and equivalent conditioning effects between groups.

## Method

### Sample

A total of 48 undergraduate students (age: mean [M] = 22.8, standard deviation [SD] = 2.9 years; gender: 38 females, 10 males; handedness: 46 right, 2 left) took part in the study and were compensated either with course credit or with € 20. Individuals could participate if they were aged 18 to 30 years, had normal or corrected-to-normal vision, and reported no preexisting neurological, cardiovascular, or psychological disorders. Individuals who had participated in one of our previous studies on imagery-based conditioning (Mueller et al., 2019) were not allowed to participate. Participants completed a series of personality questionnaires, including the German version (Ostendorf & Angleitner, 1993) of the Zuckerman-Kuhlman Personality Questionnaire (Zuckerman et al., 1993), to assess trait anxiety via the Neuroticism-Anxiety scale (NAnx). Informed consent for participation as well as for publishing and sharing the anonymized data was obtained from all participants at the start of the test session. The present study was conducted in accordance with the declaration of Helsinki and approved by the local ethics committee of the psychology department at University of Marburg.

### Experimental groups

At the beginning of the experiment, participants were randomly assigned to an imagery-based fear conditioning group (*n* = 24) or a classical fear conditioning group (*n* = 24). Self-reported gender (male vs. female) was counterbalanced across groups and handedness was equally distributed (1 left-handed participant in each group). The two groups were similar in terms of age (imagery-based: *M* = 22.8 years, *SD* = 2.9 years; classical: *M* = 22.8 years, *SD* = 3.2 years; *d* = −0.01) and trait anxiety (NAnx; imagery-based: *M* = 11.21, *SD* = 3.48; classical: *M* = 11.62, *SD* = 3.76; *d* = −0.12).

#### **Imagery-based fear conditioning group**

Participants in this group first underwent imagery training. They were instructed to 1) imagine receiving an electric shock on the left forearm whenever they see one particular cue, which was either a red square, a blue triangle, or a yellow circle (permutated assignment), 2) imagine a mild vibration on the left forearm whenever they see another one of the three different cues, or 3) not to think of anything particular whenever they see the third cue. Assignment of cues to imagery content was balanced across participants. Standardized imagery scripts for the shock and the vibration were provided via audio recording and involved imagery across different modalities. For electric shocks, participants had to imagine pain that “is extremely uncomfortable and barely tolerable” and how their “muscles are cramping.” Meanwhile, for vibrations, they had to imagine a sensation that “is not at all uncomfortable, and is easily tolerable” and how their “muscles are relaxed” (detailed scripts in Mueller et al., 2019). Participants had to verbally report the correct cue-imagery assignments before the conditioning paradigm started.

#### Classical fear conditioning group

In the classical fear conditioning group, electric shock US were applied via a D7A direct current stimulator (Digitimer Ltd, Hertfordshire, UK) to the left forearm. The shock stimulus had a duration of 500 ms and consisted of 25 square wave pulses of 2 ms each. In a standardized work-up procedure in the beginning of the test session, the current strength was set to a level that was “hard to tolerate” for each participant. To reduce habituation to the shock during fear acquisition, a series of five shocks was applied at the end of the workup. Participants rated the unpleasantness of each presentation on a 11-point scale (0 = not unpleasant at all; 10 = extremely unpleasant). Current strength of the first shock presentation was set to the level that previously had been determined as “hard to tolerate” and subsequently was increased whenever unpleasantness ratings dropped below the initial rating. If the last rating was below the initial one, a maximum of two additional shocks were applied. The average current strength was 0.86 mA (SD = 0.38 mA, range = 0.2–1.8 mA), and the average subjective unpleasantness level of the final shock was 8.25 (SD = 1.45, range = 5–10). The vibration US was also applied to the left forearm, delivered via a custom-build apparatus and had a duration of 500 ms. The strength was comparable to a vibrating cell phone. For habituation purposes, the vibration US was presented five times (between 8 and 12 s apart) directly before the start of the fear conditioning paradigm.

#### **Fear conditioning paradigm**

**Fig. 1:**
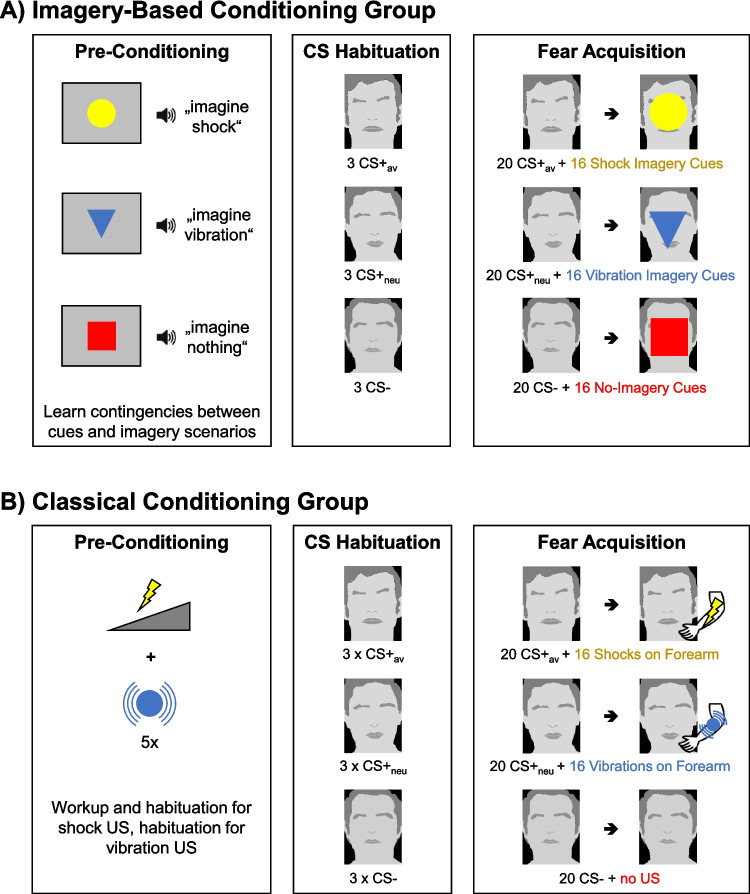
Experimental protocols for both conditioning groups. (**A**) Participants in the imagery-based conditioning group learned to imagine an electric shock, a vibration, or nothing whenever prompted by an associated cue (cues and imagery content counterbalanced). After a CS habituation phase, each CS co-terminated in 80% of the trials with one of the cues. No direct instructions on CS-imagery associations were given. (**B**) Participants in the classical conditioning group underwent a workup procedure in which the level of the electric shock US on the arm was set to a level that was perceived as hard to tolerate. They also received five presentations of the vibration US on their arm for habituation purposes. After CS habituation, two of the CS (CS+_av_, CS+_neu_) were paired in 80% of the trials with the aversive shock US and the neutral vibration US, respectively, whereas the CS− was never followed by a US. For the experiment, we used original stimuli from the Ekman face set as CS. They are not shown here due to copyright restrictions

In both groups, three faces from the Ekman series (Ekman & Friesen, 1976) were used as three different CS (CS+_av_, CS+_neu_, and CS−; see next paragraph for explanation). The assignment of faces to CS types was counterbalanced across participants. In the initial habituation phase, each of the CS was presented three times in random order. The subsequent fear acquisition phase contained two blocks with ten CS presentations each, again in randomized order (Fig. 1).

In the imagery-based fear conditioning group, the CS+_av_ was paired in 80% of the trials with the cue for the aversive imagery (electric shock), the CS+_neu_ was paired in 80% of the trials with the cue for neutral imagery (vibration), and the CS− was paired in 80% of the trials with the cue to think of nothing in particular (Fig. 1A). Conditioned stimuli were presented for a total of 10 s and, in paired trials, co-terminated with the cue, which was superimposed on the face for 3 s starting 7 s after CS onset.

In the classical fear conditioning group, the CS+_av_ was paired in 80% of the trials with an actual electric shock, the CS+_neu_ was paired in 80% of the trials with an actual vibration, and the CS− was never paired with any event (Fig. 1B). Here, CS were presented for 7.5 s and, in paired trials, co-terminated with the 0.5 s shock or vibration US, which started 7 s after CS onset.

We included imagery/application of a vibration stimulus as an active control to test whether conditioned responses are potentiated specifically because of the aversive nature of the US or merely because of *some* US expectation. Note that there were different CS and US durations in the imagery-based and the classical fear conditioning group. We chose the shorter duration of 0.5 s for physical US, because this is a common setting in fear conditioning (Sperl et al., 2016) and avoids overstimulation of participants. At the same time, this duration would be too short for participants to reliably produce imagery (Mueller et al., 2019). This resulted in different trial durations for classical and imagery-based fear conditioning groups but allowed us to use the same CS-US interval of 7 s in both groups. In both groups, trials were separated by an intertrial interval with jittered duration (8,000–10,000 ms randomly drawn from uniform distribution) during which a white fixation cross was presented.

### Ratings

All participants rated the face CS on 5-point Likert scales with regard to experienced unpleasantness (“How pleasant or unpleasant do you find this face on a scale from 1 to 5?” with labels for the scale extremes: 1 = very pleasant, 5 = very unpleasant), arousal (“How arousing do you find this face on a scale from 1 to 5?”; 1 = not arousing, 5 = very arousing), fear/anxiety, anger, and disgust (“How [anxious/angry/disgusted] are you seeing this face on a scale from 1 to 5?”; 1 = not [anxious/angry/disgusted], 5 = very [anxious/angry/disgusted]). Conditioned stimulus ratings were collected four times: before CS habituation as well as before, midway through, and after acquisition. Participants also rated the unpleasantness of the imagery contents (imagery-based conditioning group) or of the physical US (classical conditioning group) on an 11-point Likert scale before habituation, between the two acquisition blocks, and at the end of the acquisition phase.

### Physiological recordings and preprocessing

All physiological data were recorded at 1024 Hz with a BioSemi ActiveTwo system (BioSemi, Amsterdam, Netherlands). A Bessel lowpass filter (fifth order, −3 dB at 204.8 Hz) was applied online.

#### Skin conductance response

Skin conductance was measured at the thenar and hypothenar of the left hand with exosomatic measurement (16 Hz AC at 1 µA). In BrainVision Analyzer 2 (Brain Products, Gilching, Germany), skin conductance data were lowpass filtered (−3 dB at 1 Hz, 24 dB/oct., zero-phase IIR Butterworth filter) and manually screened for artifacts. Segments were extracted from −1 to 5 s relative to CS onset. Using a custom MATLAB (Mathworks, Natick, MA) script, single-trial data were baseline-corrected (−1 to 0 s), peaks were detected in the time window from 1 to 5 s, and resulting amplitudes were normalized, dividing by the largest absolute response across all trials of the same participant (Lykken & Venables, 1971). Individual SCRs were then computed by averaging the normalized single-trial peaks for each participant and CS.

#### Interbeat interval

Electrocardiogram was recorded in Lead II configuration (left leg, right forearm). In BrainVision Analyzer 2, the electrocardiogram signal was bandpass-filtered (−3 dB at 0.1/30 Hz, 24 dB/oct., zero-phase IIR Butterworth filter) and notch-filtered (50 Hz, 5 Hz bandwidth, 16th order). R spikes were detected automatically (EKG Marker solution) and markers were corrected manually if necessary. Data segments were excluded manually from analysis if they contained premature systoles or artifacts masking R spikes. Using the MATLAB solution in BrainVision Analyzer 2 and a custom MATLAB script, we computed a continuous IBI trace with each sample indicating the distance between preceding and subsequent R spike in milliseconds (Mueller et al., 2013). Segments (−1 to 7 s relative to CS) were extracted from the continuous signal, baseline-corrected (−1 to 0 s), and averaged across trials for each participant and condition. Based on previous conditioning studies (Mueller et al., 2019; Panitz et al., 2015; 2018; Sperl et al., 2016), the time window from 2 to 5 s was preregistered and used to compute average IBI responses for statistical analyses.

#### **Late positive potential**

Electroencephalogram (EEG) was assessed with 64 electrodes positioned according to the 10–20 system, with Common Mode Sense and Driven Right Leg electrodes at parietal sites. In BrainVision Analyzer 2, the EEG signal was referenced against the average. Electroencephalogram was corrected for eye-induced artifacts via independent component analysis (ICA; extended infomax algorithm). To reduce computation time for ICA, data were first downsampled to 512 Hz. A high-pass filter (−3 dB at 1 Hz, 24 dB/oct., zero-phase IIR Butterworth filter) was applied to increase stationarity of the EEG signal and thereby improve the ICA solution. The resulting ICA weights were applied to EEG (at 1024 Hz) that was highpass filtered with 0.1 Hz in order to preserve the slow LPP. This approach has been validated (Winkler et al., 2015) and has been successfully used by our lab in previous studies (Panitz et al., 2018; 2019). For the 0.1 Hz filtered data, independent components reflecting blinks and eye movement were identified based on signal shape and topography, and removed from the EEG data. Electroencephalogram data were screened by trained raters. Channels with excessive amounts of bad data were interpolated (spline interpolation) and data was again referenced to the new average. Data with remaining artifacts were excluded manually by trained raters, supervised by the first author. The remaining data were segmented relative to CS (−200 to 1,000 ms), baseline-corrected (−200 to 0 ms), and referenced against the average of P9 and P10 (mastoid reference). Mean amplitude at Pz in the preregistered time window from 300 to 700 ms post-CS (also see Panitz et al., 2015) was used for statistical analyses on the LPP. Comparable amounts of trials were available across conditions in the imagery-based conditioning group (CS+_av_: *M* = 17.3, *SD* = 2.2; CS+_neu_: *M* = 16.8, *SD* = 1.8; CS−: *M* = 17.7, *SD* = 1.7) and in the classical conditioning group (CS+_av_: *M* = 15.8, *SD* = 3.8; CS+_neu_: *M* = 16.3, *SD* = 3.5; CS−: *M* = 16.5, *SD* = 3.4). Note that, in addition to preregistered analyses, we also conducted exploratory LPP analyses for the window from 400 to 1,000 ms, averaging across Pz, POz, P3, P1, P2, P4, PO3, PO4 and using an average reference.

### Statistical analyses

Statistical analyses were conducted by using R 4.3.1 (R Core Team, 2020) in the RStudio 2023.06.0 environment (RStudio Team, 2016). Both frequentist (α = 0.05) and Bayesian test statistics were computed.

For all dependent variables and separately for the two conditioning groups (imagery-based and classical), we computed Analyses of Variance (ANOVA) with the factor CS Type (CS+_av_, CS+_neu_, CS−). We followed up with pairwise comparisons of the CS. More specifically, we computed one-sided *t*-tests for directed hypotheses (CS+_av_ > CS+_neu_, CS+_av_ > CS−) and two-sided *t*-tests for the exploratory comparison between CS+_neu_ and CS−. Second, we computed a Conditioning Group x CS Type ANOVA and followed up with exploratory two-sample *t*-tests of differential responses (two-sided, Bonferroni-corrected) in case of a significant interaction term. For frequentist ANOVAs (ezANOVA function of the ez package), type III sums of squares were used, and *p*-values were Greenhouse-Geisser corrected (*p*_GG_) if the Mauchley test was significant. For frequentist *t*-tests, the t.test function of the stats package was used, and *p*-values were corrected by using the Welch modification of degrees of freedom. For Bayesian ANOVAs, the anovaBF function from the BayesFactor package was used and 100,000 iterations for Monte Carlo Simulation of the g priors were chosen (Rouder et al., 2012). For two-way ANOVAs, Bayes factors were computed for all submodels (i.e., all combinations of main and interaction effects; equal prior probabilities) and with the null model including the grand mean and participants’ means as random intercepts. Inclusion Bayes Factors were computed for each effect (Hinne et al., 2020), comparing all models including this effect with the remaining models, not including the effect (Makowski et al., 2019). For follow-up *t*-tests, we used the ttestBF function of the BayesFactor package, which places a Cauchy prior on the standardized effect size (Rouder et al., 2009). To conduct one-sided tests, prior probabilities were set to zero for effect size values opposing the predicted direction. For one-factorial ANOVAs and *t*-tests, Bayes factors in this study always indicate evidence of the given model over the null model (BF_10_).

### Preregistration

Central characteristics of this study and the analyses had been preregistered on OSF (https://osf.io/sygd6). This includes the sample size, the experimental design, the time windows, and other specifications for the physiological responses (mastoid reference for EEG, normalization for SCR) and statistical analyses. All analyses have been conducted as described in the preregistration with one exception: we provide Bayesian statistics for the within-group ANOVAs and *t*-tests, although they were only preregistered for the two-way ANOVA. In order to streamline the manuscript, we only report analyses of fear ratings in the main manuscript. Preregistered analyses for unpleasantness and arousal ratings indicated successful fear conditioning in both groups and are reported in detail in Supplement 1, together with analyses of anger and disgust ratings.

We also conducted preregistered supplementary analyses. First, we repeated the main analyses using only contingency-aware participants (*n* = 22 in both groups; Supplement 4). There is no clear consensus in the fear conditioning literature as to whether participants without contingency awareness should be included in analyses (Lonsdorf et al., 2017). In order to be transparent about potential biases because of data inclusion vs. exclusion (Lonsdorf et al., 2017; 2019), we conducted these additional analyses. Second, we had preregistered supplementary analyses on the changes in conditioned responses across the experiment adding the factor Time for rating data (pre-acquisition, mid-acquisition, post-acquisition) and physiological data (first half, second half; Supplement 5) in case that conditioning effects do not occur before the second half of the paradigm. Third, while not the focus of the current study, we analyzed frontomedial theta from 0 to 2 s post-CS, which we found sensitive to conditioning in a previous publication using a classical fear conditioning design with physical US (Mueller et al., 2014). Finally, we decided against preregistered analyses on trait measures of imagery vividness in the imagery-based conditioning groups given that the sample size is not adequate to test personality effects (Mar et al., 2013).

We conducted two sets of analyses that were not preregistered. First, to gain an additional perspective on skin conductance data beyond amplitudes, we also computed the number of trials per participant and condition in which an SCR was present at all, using a 0.05 µS threshold as criterion (Boucsein et al., 2012; Lonsdorf et al., 2017). These analyses converged with results on SCR amplitudes and are reported in Supplement 2. Moreover, we conducted analyses for unpleasantness ratings, SCR, IBI, and ERPs to the imagery cues to get deeper insights into the mechanisms of imagery-based conditioning. Here, higher unpleasantness ratings as well as increased SCR amplitudes and cardiac acceleration were observed for the CS+_av_, replicating previous findings (Mueller et al., 2019). Detailed methods and analyses can be found in Supplement 6. In the process of analyzing cue-evoked LPP, topographies also suggested a cue-related modulation of a sustained frontocentral negativity. Analyses on cue-evoked ERPs are reported at the end of the *Results* section.

### Open materials

We provide the Presentation code for the stimulus presentation (with exception of the pictures from the Ekman series due to copyright), as well as data and R scripts for statistical analyses on the Open Science Framework (https://osf.io/f36jb). Raw physiological data are available upon request.

## Results

### Ratings

**Fig. 2:**
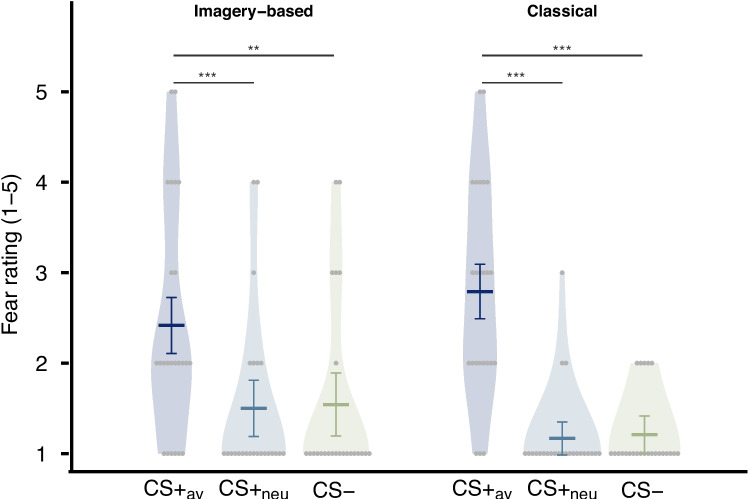
Individual (dots) and mean (horizontal bars) subjective fear ratings (1 = not fearful at all, 5 = very fearful) at the end of the experiment for each CS and separate for the imagery-based and the classical conditioning group. Error bars depict the 95% confidence interval of the mean based on within-subject variance. **p <.01; ***p <.001

The Conditioning Group x CS Type ANOVA on fear ratings of the face CS revealed a significant main effect for CS Type (*F*(2, 92) = 33.23,* p* <.001, η_p_^2^ =.419, *BF*_*Incl*_ = 7.01 e+9). More specifically, fear ratings in response to CS+_av_ were elevated relative to CS+_neu_ (*t*(47) = 7.04, *p*_*one-tailed*_ <.001, *d* = 1.02, *BF*_*10, one-tailed*_ = 3.51 e+6) and CS− (*t*(47) = 6.21, *p*_*one-tailed*_ <.001, *d* = 0.90, *BF*_*10, one-tailed*_ = 3.51 e+6), while there was basically no difference between CS+_neu_ and CS− fear ratings (*t*(47) = −0.26, *p*_*two-tailed*_ =.796, *d* = −0.04, *BF*_*10, two-tailed*_ = 0.16). There was no significant main effect of Conditioning Group (*F*(2, 46) = 0.29, *p* =.592, η_p_^2^ =.006, *BF*_*Incl*_ = 0.26). Importantly, the Conditioning Group x CS Type interaction did not reach significance (*F*(2, 92) = 2.67, *p* =.075, η_p_^2^ =.055, *BF*_*Incl*_ = 1.01). Fear rating results are depicted in Fig. 2.

#### Imagery-based conditioning group

In the imagery-based conditioning group, an ANOVA on fear ratings showed a significant main effect of CS Type (*F*(2, 46) = 6.55,* p* =.003, η_p_^2^ =.222, *BF*_*10*_ = 20.8). Fear ratings were higher for CS+_av_ compared with CS+_neu_ (*t*(23) = 3.50, *p*_*one-tailed*_ <.001, *d* = 0.71, *BF*_*10, one-tailed*_ = 39.6) and for CS+_av_ compared with CS− (*t*(23) = 2.95, *p*_*one-tailed*_ =.004, *d* = 0.60, *BF*_*10, one-tailed*_ = 12.7). There was no significant difference between CS+_neu_ and CS− (*t*(23) = −0.14, *p*_*two-tailed*_ =.890, *d* = −0.03, *BF*_*10, two-tailed*_ = 0.22).

#### Classical conditioning group

In the classical conditioning group, the ANOVA on fear ratings showed a significant main effect of CS Type (*F*(2, 46) = 39.25, *p* <.001, η_p_^2^ =.631, *BF*_*10*_ = 5.28 e+9). Fear ratings were higher for CS+_av_ compared with CS+_neu_ (*t*(23) = 7.01, *p*_*one-tailed*_ <.001, *d* = 1.43, *BF*_*10, one-tailed*_ = 87,661) and for CS+_av_ compared with CS− (*t*(23) = 6.40, *p*_*one-tailed*_ <.001, *d* = 1.31, *BF*_*10¸one-tailed*_ = 23,414). There was no significant difference between CS+_neu_ and CS− (*t*(23) = −0.33, *p*_*two-tailed*_ =.747, *d* = −0.07, *BF*_*10, two-tailed*_ = 0.23).

Taken together, we found successful conditioning of fear ratings both in imagery-based and classical conditioning. There were no significant differences in the magnitude of these effects between conditioning groups.

### Skin conductance responses

**Fig. 3:**
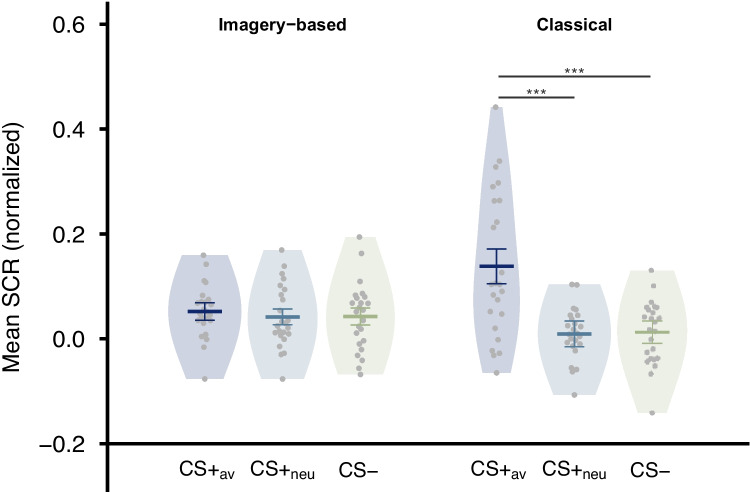
Individual (dots) and mean (horizontal bars) normalized skin conductance response for each CS and separate for the imagery-based and the classical conditioning group. Error bars depict the 95% confidence interval of the mean based on within-subject variance. ***p <.001

The Conditioning Group x CS Type ANOVA revealed a significant main effect for CS Type (*F*(2, 92) = 16.53, *p* <.001, η_p_^2^ =.264, *BF*_*Incl*_ = 41,089) with elevated SCR amplitudes to CS+_av_ compared with CS+_neu_ (*t*(47) = 3.94, *p*_*one-tailed*_ <.001, *d* = 0.57, *BF*_*10, one-tailed*_ = 190.48) and compared with CS− (*t*(47) = 4.00, *p*_*one-tailed*_ <.001, *d* = 0.58, *BF*_*10, one-tailed*_ = 222.67). SCR amplitudes to CS+_neu_ and to CS− were comparable (*t*(47) = −0.19, *p*_*two-tailed*_ =.849, *d* = −0.03, *BF*_*10, two-tailed*_ = 0.16). Meanwhile, no significant main effect of Conditioning Group emerged (*F*(2, 46) = 0.26, *p* =.615, η_p_^2^ =.006, *BF*_*Incl*_ = 0.27). Importantly, the Conditioning Group x CS Type interaction reached significance (*F*(2, 92) = 12.06, *p* <.001, η_p_^2^ =.208, *BF*_*Incl*_ = 1,416). This was driven by the pattern that the classical conditioning group showed stronger differential responses for both CS+_av_ compared with CS+_neu_ (*t*(46) = 3.81, *p*_*Bonferroni*_ =.002, *d* = 1.10, *BF*_*10, two-tailed*_ = 65.0) and CS+_av_ compared with CS− (*t*(46) = 3.92, *p*_*Bonferroni*_ =.001, *d* = 1.13, *BF*_*10, two-tailed*_ = 87.6). Meanwhile the lack of differentiation between CS+_neu_ and CS− was comparable in the two groups (*t*(46) = −0.11, *p*_*Bonferroni*_ = 1, *d* = −0.03, *BF*_*10, two-tailed*_ = 0.29). Skin conductance response results are presented in Fig. 3.

#### **Imagery-based conditioning group**

In the imagery-based conditioning group, the one-factorial ANOVA showed no significant effect of CS Type on SCR (*F*(2, 46) = 0.34,* p* =.717, η_p_^2^ =.014, *BF*_*10*_ = 0.15; Fig. 3), which is consistent with the lack of effects on SCR in our prior publication on imagery-based fear conditioning (Mueller et al., 2019). In line with the ANOVA, pairwise comparisons provided some evidence for comparable SCRs across CS (all |*t*|(23) < 0.75, *p* >.232, |*d*| < 0.16, *BF*_*10*_ < 0.43).

#### **Classical conditioning group**

Unlike in the imagery-based conditioning group, the ANOVA in the classical conditioning group showed a strong, significant effect of CS Type on SCR (*F*(2, 46) = 19.27, *p* <.001, η_p_^2^ =.456, *BF*_*10*_ = 82,370). This effect was driven by higher SCR amplitudes to CS+_av_ both compared with CS+_neu_ (*t*(23) = 4.64, *p*_*one-tailed*_ <.001, *d* = 0.95, *BF*_*10, one-tailed*_ = 480) and compared with CS− (*t*(23) = 4.91, *p*_*one-tailed*_ <.001, *d* = 1.00, *BF*_*10, one-tailed*_ = 886). Meanwhile, CS+_neu_ and CS− evoked comparable SCR amplitudes (*t*(23) = −0.20, *p*_*two-tailed*_ =.845, *d* = −0.04, *BF*_*10, two-tailed*_ = 0.22).

Taken together, we found robust fear conditioning effects of the SCR in the classical conditioning group. Meanwhile, in line with previous samples, we found substantial Bayesian evidence for the null hypothesis in the imagery-based conditioning group, that is, for the absence of conditioning effects on SCR. This pattern was replicated when analyzing the number of nonzero responses rather than the average SCR amplitude as reported in Supplement 2.

### Interbeat interval

The Conditioning Group x CS Type ANOVA showed stronger cardiac slowing in classical conditioning in general whereas net cardiac acceleration was observed in imagery-based conditioning (main effect Conditioning Group: *F*(1, 46) = 8.06, *p* =.007, η_p_^2^ =.149, *BF*_*Incl*_ = 5.13). Across groups, there was a significant main effect of CS Type (*F*(2, 46) = 3.77, *p* =.027, η_p_^2^ =.076, *BF*_*Incl*_ = 1.90) indicating increased cardiac slowing to the CS+_av_ vs. CS− across groups (*t*(47) = 2.77, *p*_*one-tailed*_ =.004, *d* = 0.40, *BF*_*10, one-tailed*_ = 9.16). There were no significant differences between CS+_av_ and CS+_neu_ (*t*(47) = 1.06, *p*_*one-tailed*_ =.147, *d* = 0.15, *BF*_*10,*__*one-tailed*_ = 0.45) or between CS+_neu_ and CS− (*t*(47) = 1.65, *p*_*two-tailed*_ =.105, *d* = 0.24, *BF*_*10, two-tailed*_ = 0.56). ANOVA results suggested no differing effects of CS Type across conditioning groups (CS Type x Conditioning Group interaction: *F*(2, 92) = 0.21, *p* =.808, η_p_^2^ =.005, *BF*_*Incl*_ = 0.14).

#### **Imagery-based conditioning group**

The one-factorial ANOVA did not show a significant effect of CS Type on evoked IBI in the imagery-based conditioning (*F*(2, 46) = 1.19, *p* =.313, η_p_^2^ =.033, *BF*_*10*_ = 0.34). Consequently, pairwise comparisons showed only a trend toward significance for slower heart beat after CS+_av_ compared with CS− (*t*(23) = 1.57, *p*_*one-tailed*_ =.065, *d* = 0.32, *BF*_*10, one-tailed*_ = 1.16).

#### **Classical conditioning group**

The effect of CS Type on IBI did not quite reach significance in the classical conditioning either (*F*(2, 46) = 2.87, *p* =.067, η_p_^2^ =.053, *BF*_*10*_ = 1.06). One-sided pairwise comparisons showed more cardiac slowing for CS+_av_ compared with CS− (*t*(23) = 2.33, *p*_*one-tailed*_ =.015, *d* = 0.47, *BF*_*10, one-tailed*_ = 3.93). There were no significant differences between CS+_av_ and CS+_neu_ (*t*(23) = 0.62, *p*_*one-tailed*_ =.272, *d* = 0.13, *BF*_*10, one-tailed*_ = 0.37) or between CS+_neu_ and CS− (*t*(23) = 1.77, *p*_*two-tailed*_ =.090, *d* = 0.36, *BF*_*10, two-tailed*_ = 1.57).

**Fig. 4:**
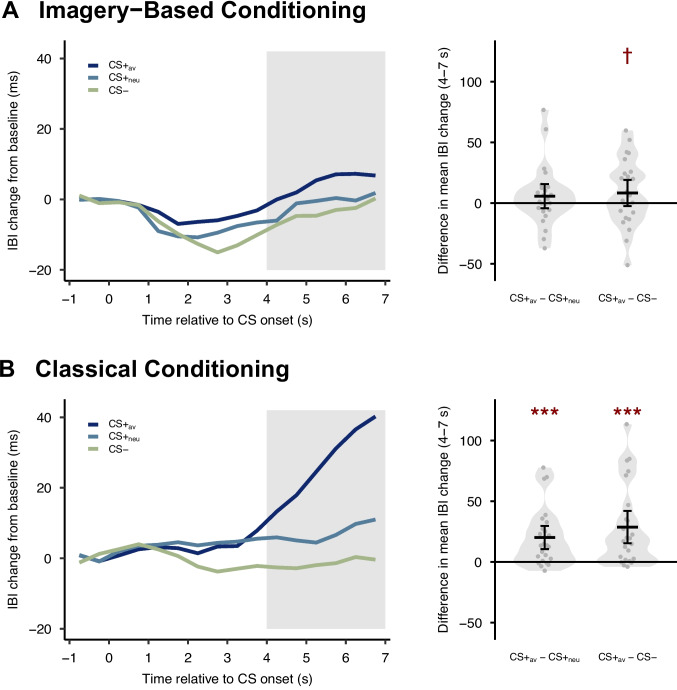
Interbeat interval (IBI) time course in response to the different CS and differences in mean evoked IBI from 4 to 7 s post-CS (gray area in time course) for the comparisons [CS+_av_ – CS+_neu_] and [CS+_av_ – CS−]. Individual values are shown as dots, the group means are shown as horizontal bars. Error bars depict the 95% confidence interval of the mean based on within-subject variance. ***p <.001; ^†^*p* <.10. (**A**) Evoked IBI in the imagery-based conditioning group. (**B**) Evoked IBI in the classical conditioning group

*Exploratory analyses for IBI from 4 to 7 s.* Visual inspection of the IBI time courses suggested that the a priori time window of 2 to 5 s was not ideal to capture effects of CS Type. We therefore repeated analyses using a shifted time window from 4 to 7 s. This did not change the pattern in the imagery-based conditioning group, because there was still no significant main effects of CS Type (*F*(2, 46) = 1.15, *p* =.326, η_p_^2^ =.048, *BF*_*Incl*_ = 0.37) and only a trend for more deceleration for CS+_av_ compared with CS− (*t*(23) = 1.52, *p*_*one-tailed*_ =.071, *d* = 0.31, *BF*_*10, one-tailed*_ = 1.08). Meanwhile, the classical conditioning group now showed a significant main effect of CS Type (*F*(2, 46) = 12.98, *p* <.001, η_p_^2^ =.361, *BF*_*Incl*_ = 2,677) mirroring prior work on fear-conditioned cardiac slowing (Panitz et al., 2015, 2018; Sperl et al., 2016). This was caused by stronger cardiac deceleration for CS+_av_ compared with CS+_neu_ (*t*(23) = 4.13, *p*_*one-tailed*_ <.001, *d* = 0.84, *BF*_*10, one-tailed*_ = 156.1) and compared with CS− (*t*(23) = 4.24, *p*_*one-tailed*_ <.001, *d* = 0.87, *BF*_*10, one-tailed*_ = 197.3), but there was no significant difference between CS+_neu_ and CS− (*t*(23) = 1.55, *p*_*two-tailed*_ =.136, *d* = 0.32, *BF*_*10, two-tailed*_ = 0.61). In line with increased effects in the classical conditioning group, the Conditioning Group x CS Type interaction was significant for the later time window (*F*(2, 92) = 3.36, *p* =.039, η_p_^2^ =.068, *BF*_*Incl*_ = 2.27). This was driven by increased differential responses in the classical conditioning group both for CS+_av_ compared with CS+_neu_ (*t*(46) = 2.05, *p*_*Bonferroni*_ <.046, *d* = 0.59, *BF*_*10, two-tailed*_ = 1.54) and for CS+_av_ compared with CS− (*t*(46) = 2.33, *p*_*Bonferroni*_ <.024, *d* = 0.67, *BF*_*10, two-tailed*_ = 2.47). Interbeat interval results are shown in Fig. 4.

Taken together, there was a strong effect of fear conditioning on cardiac deceleration in the classical conditioning group when analyzing the time window immediately leading up to the US (i.e., 4 to 7 s after CS onset). Meanwhile, there was no substantial evidence for conditioned modulation of the interbeat interval in imagery-based conditioning.

### Late positive potential

Similar to IBI, we conducted exploratory analyses on LPP amplitudes. Based on inspection of ERP waveforms and topographies, we extracted LPP amplitudes as the mean amplitude in the time window from 400 to 1,000 ms, averaged across multiple posterior electrodes (Pz, POz, P1, P2, P3, P4, PO3, PO4). Moreover, we computed ERPs with an average reference as used in previous studies (Schindler et al., 2020; Suess & Rahman, 2015). While the descriptive pattern was comparable, the pairwise comparisons between CS+_av_ and CS+_neu_ was significant in the exploratory approach but only approaching significance in the preregistered analysis. Here, we report results for the later time window (400 to 1,000 ms) across multiple sites and referenced to the average while preregistered analyses are reported in Supplement 3.

**Fig. 5:**
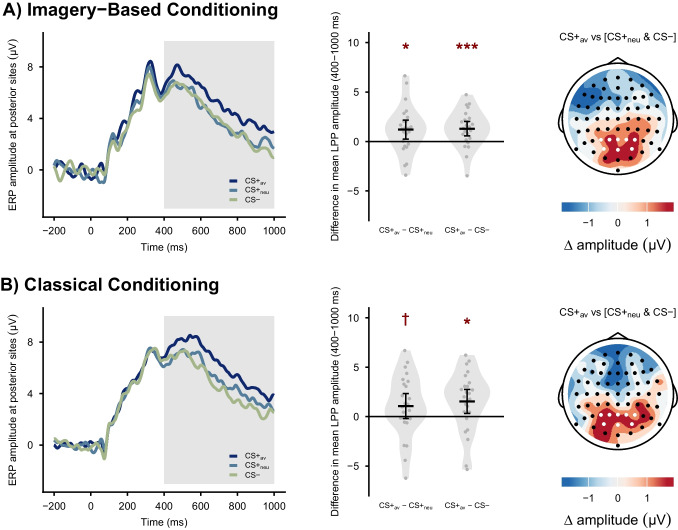
The late positive potential (LPP) at posterior electrodes (Pz, POz, P1, P2, P3, P4, PO3, PO4) in response to the different CS and separate for the imagery-based and the classical conditioning group. The shaded areas in the ERP panels indicate the exploratory time window (400 to 1,000 ms post-CS). The graphs in the middle depict differences of mean LPP amplitudes at posterior electrodes (Pz, POz, P1, P2, P3, P4, PO3, PO4) from 400 to 1,000 ms for the comparisons [CS+_av_ – CS+_neu_] and [CS+_av_ – CS−]. Individual values are shown as dots, the group means are shown as horizontal bars. Error bars depict the 95% confidence interval of the mean based on within-subject variance. ***p <.001; *p <.05; ^†^*p* <.10. Topographies show the contrast comparing CS+_av_ to the other two CS (weights: [1, −0.5, −0.5])

The Conditioning Group x CS Type ANOVA showed a significant main effect for CS Type (*F*(2, 92) = 8.37, *p* <.001, η_p_^2^ =.154, *BF*_*Incl*_ = 65.64) with elevated LPP amplitudes to CS+_av_ compared with CS+_neu_ (*t*(47) = 2.85, *p*_*one-tailed*_ =.003, *d* = 0.41, *BF*_*10, one-tailed*_ = 11.14) and compared with CS− (*t*(47) = 3.98, *p*_*one-tailed*_ <.001, *d* = 0.57, *BF*_*10, one-tailed*_ = 210.85). There was moderate evidence for equal LPP amplitudes to CS+_neu_ and to CS− (*t*(47) = 0.84, *p*_*two-tailed*_ =.403, *d* = 0.12, *BF*_*10, two-tailed*_ = 0.22). Meanwhile, no main effect of Conditioning Group on LPP amplitudes emerged (*F*(1, 46) = 1.90, *p* =.175, η_p_^2^ =.040, *BF*_*Incl*_ = 0.70). Importantly, ANOVA results suggested similar effects of CS Type across conditioning groups with moderate Bayesian evidence against the CS Type x Conditioning Group interaction (*F*(2, 92) = 0.14, *p* =.871, η_p_^2^ =.003, *BF*_*Incl*_ = 0.13). In line with the lack of an interaction effect, none of the pairwise comparisons of differential responses between groups reached significance (all |*t*| < 0.58, *p*_*Bonferroni*_ = 1, |*d*| < 0.17, *BF*_*10*_ < 0.33). Late positive potential results are shown in Fig. 5.

#### **Imagery-based conditioning group**

The main effect of CS Type was significant for the LPP in the imagery-based conditioning group (*F*(2, 46) = 5.67, *p* =.006, η_p_^2^ =.198, *BF*_*10*_ = 6.69). In line with our preregistered hypotheses, LPP amplitudes showed a significant increase for CS+_av_ compared with CS− (*t*(23) = 3.49, *p*_*one-tailed*_ <.001, *d* = 0.71, *BF*_*10, one-tailed*_ = 38.35) and for CS+_av_ compared with CS+_neu_ (*t*(23) = 2.48, *p*_*one-tailed*_ =.010, *d* = 0.51, *BF*_*10, one-tailed*_ = 5.17). There was no significant difference in LPP amplitudes between CS+_neu_ and CS− (*t*(23) = 0.21, *p*_*two-tailed*_ =.833, *d* = 0.04, *BF*_*10, one-tailed*_ = 0.22).

#### **Classical conditioning group**

The effect of CS Type on LPP was significant in the classical conditioning group as well (*F*(2, 46) = 3.51, *p* =.038, η_p_^2^ =.133, *BF*_*10*_ = 1.58). One-sided pairwise comparisons showed more positive LPP amplitudes for CS+_av_ compared with CS− (*t*(23) = 2.50, *p*_*one-tailed*_ =.010, *d* = 0.51, *BF*_*10, one-tailed*_ = 5.37) but not quite compared with CS+_neu_ (*t*(23) = 1.66, *p*_*one-tailed*_ =.055, *d* = 0.34, *BF*_*10, one-tailed*_ = 1.33). There was no significant difference between LPP amplitudes evoked by the CS+_neu_ compared with the CS− (*t*(23) = 0.91, *p*_*two-tailed*_ =.374, *d* = 0.19, *BF*_*10, two-tailed*_ = 0.31).

### Exploratory analyses on cue-locked responses in imagery-based conditioning

To better understand the mechanisms of imagery-based conditioning, we analyzed unpleasantness ratings of each imagery, as well as SCR, IBI, and ERPs time-locked to the cue onset. Analyses of ratings revealed that imagery prompted by the aversive cue was perceived as significantly more unpleasant than imagery prompted by the neutral imagery cue or the no-imagery cue. Moreover, we found increased SCR amplitudes and stronger cardiac acceleration in response to the aversive imagery cue compared with both the neutral and the no-imagery cue, replicating previous findings (Mueller et al., 2019). Detailed results of ratings, SCR, and IBI to cues/imagery can be found in Supplement 6.

**Fig. 6:**
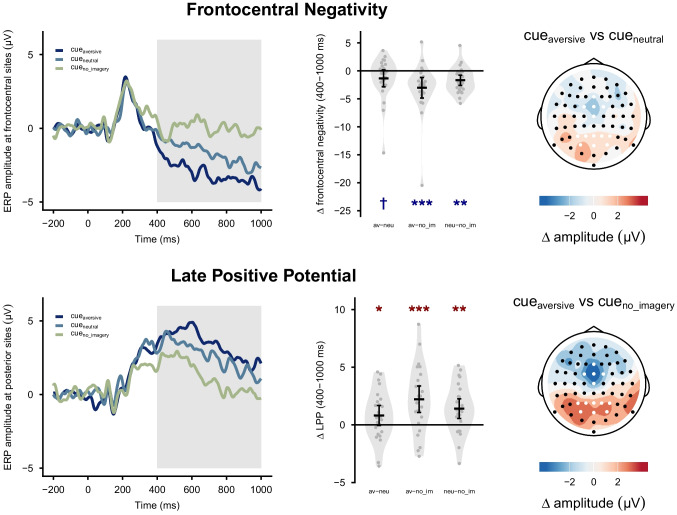
Event-related potentials at frontocentral (Fz, FC1, FCz, FC2, Cz) and posterior electrodes for LPP measurement (Pz, POz, P1, P2, P3, P4, PO3, PO4) in response to imagery cues in the imagery-based conditioning group. The shaded areas in the ERP panels indicate the time window for averaging (400 to 1,000 ms post-CS). The violin graphs in the middle depict differences between cues in mean ERP amplitudes at frontocentral and posterior electrodes from 400 to 1,000 ms. Individual values are shown as dots, the group means are shown as horizontal bars. Error bars depict the 95% confidence interval of the mean based on within-subject variance. ***p <.001; **p <.01; *p <.05; ^†^*p* <.10. *p*-values are one-sided for [av-neu] and [av-no_im] for LPP analyses, two-sided otherwise. The topographies show the contrasts between cue_aversive_ and cue_neutral_, as well as between cue_aversive_ and cue_no_imagery_

For the LPP, we used the same preprocessing stream as for CS-evoked ERPs and segmented data from −200 to 1,000 relative to cue onsets. In contrast to the preregistration, the ERP was re-referenced to the average. In line with optimized analyses of the CS-evoked LPP, we averaged ERP amplitudes at posterior sites (Pz, POz, P1, P2, P3, P4, PO3, PO4) in the time window from 400 to 1,000 ms. The ANOVA with the within-subject factor Cue Type was significant (*F*(2, 46) = 10.40, *p* <.001, η_p_^2^ =.311, *BF*_*10*_ = 144.51; Fig. 6). This effect was qualified by larger LPP amplitudes to aversive vs. neutral cues (*t*(23) = 1.86, *p*_*one-tailed*_ =.038, *d* = 0.38, *BF*_*10, one-tailed*_ = 1.79), aversive vs. no-imagery cues (*t*(23) = 3.78, *p*_*one-tailed*_ <.001, *d* = 0.77, *BF*_*10, one-tailed*_ = 72.45), and neutral vs. no-imagery cues (*t*(23) = 3.23, *p*_*two-tailed*_ =.004, *d* = 0.66, *BF*_*10, two-tailed*_ = 11.22).

In addition, inspection of the ERP topographies (Fig. 6) suggested the existence of a sustained frontocentral negativity modulated by Cue Type. We therefore conducted exploratory analyses, computing the average amplitude at frontocentral sites (Fz, FC1, FCz, FC2, Cz) from 400 to 1,000 ms post-cue. The one-factorial ANOVA revealed a significant effect of Cue Type on the frontocentral negativity (*F*(2, 46) = 8.06, *p* =.001, η_p_^2^ =.259, *BF*_*10*_ = 36.88; Fig. 6). Two-sided follow-up *t*-tests revealed that aversive cues evoked a tentatively more negative amplitude than neutral cues (*t*(23) = −1.72, *p*_*two-tailed*_ =.098, *d* = −0.35, *BF*_*10, two-tailed*_ = 0.77) and a significantly more negative amplitude than no-imagery cues (*t*(23) = −3.22, *p*_*two-tailed*_ =.004, *d* = −0.66, *BF*_*10, two-tailed*_ = 11.14). Neutral cues still evoked a significantly more negative amplitude than no-imagery cues (*t*(23) = −3.56, *p*_*two-tailed*_ =.002, *d* = −0.73, *BF*_*10, two-tailed*_ = 22.47).

## Discussion

In the present study, we investigated subjective and electrophysiological responses in an imagery-based fear conditioning paradigm, using aversive imagery as US, in direct comparison to a classical conditioning procedure, using physical US. In particular, we were interested in 1) whether the LPP as a central marker of thorough stimulus processing is modulated by imagery-based conditioning and 2) whether different conditioned responses differ quantitatively between classical and imagery-based conditioning. With regard to the first question, we found significantly increased LPP amplitudes in imagery-based conditioning for CS+_av_ compared with CS− and with CS+_neu_. When comparing responses from classical and imagery-based conditioning with Bayesian statistics, we found comparable responses in the LPP and fear ratings. Meanwhile, significant group differences were found for peripheral autonomic measures, that is IBI and SCR, with stronger conditioned responses for classical conditioning.

The current results show that pairing a CS with an aversive mental image US increases CS-evoked LPP amplitudes. We thereby provide first evidence for the potential of imagery-based fear conditioning to modulate cortical responses of stimulus processing, beyond previous studies using ratings and peripheral autonomic measures. While such rating and autonomic data showed that imagery-based conditioning can establish completely new CS-US associations (Mueller et al., 2019), the identification of the LPP as a sensitive electrocortical component advances our understanding of the underlying mechanisms. Higher amplitudes in the LPP, which is generated from the reciprocal interplay between prefrontal and occipital cortices (Moratti et al., 2011), are thought to reflect perceptual and attentional facilitation, as well as thorough post-perceptual processing of motivationally salient stimuli (Moratti et al., 2011; Schupp et al., 2000; Wieser & Keil, 2020). Therefore, the present data provide evidence that CS associated with aversive imagery acquire more motivational relevance and are processed more thoroughly than CS not predictive of aversive experiences—as has been shown in classically conditioned CS (Bacigalupo & Luck, 2022; Panitz et al., 2019). With regard to the neural basis of such stimulus processing, the LPP has been shown to be modulated by activity in several regions, including amygdala, anterior cingular cortex, and insula (Liu et al., 2012)—all of which play central roles in classical fear conditioning (Fullana et al., 2016; Sperl et al., 2019). Whether the cortico-limbic network identified in classical conditioning has the same relevance for imagery-based conditioning should be investigated in future studies using MRI.

In order to better understand the mechanisms of imagery-based conditionings on central stimulus processing, we also conducted exploratory analyses on cue-evoked ERPs. Aversive imagery cues evoked higher LPP amplitudes compared with the other cues, consistent with previous studies reporting increased LPP amplitudes in response to cued affective vs. neutral imagery (MacNamara, 2018; Schindler et al., 2020; Suess & Rahman, 2015). It is noteworthy that the present study used somatosensory imagery to induce threat, whereas previous studies used visual imagery (Suess & Rahman, 2015) or multisensory imagery with critical visual components (MacNamara, 2018; Schindler et al., 2020). Given that the LPP is widely regarded as a marker of *visual* stimulus processing (Moratti et al., 2011), LPP modulation observed here may therefore more likely reflect acquired affective qualities of the visual cues on the screen rather than imagery itself. Future conditioning studies might test this hypothesis using auditory imagery cues and/or visual imagery. The finding that the neutral imagery cue still evoked higher LPP amplitudes than the no-imagery cue may be explained by the LPP’s sensitivity to whether participants are required to respond to a stimulus (Schindler & Straube, 2020), because the no-imagery cue did not require any further (mental) action.

In addition to the LPP, imagery cues evoked and modulated a sustained frontocentral negativity, most pronounced at FCz. This effect was primarily characterized by more negative amplitudes for both imagery cues compared with the no-imagery cue, although there was a tendency toward more negative amplitudes in the aversive compared with the neutral imagery cue as well. While such frontocentral negativities are not common markers in fear conditioning research, similar waveforms have been reported during working-memory retention, where they scale with working-memory load (McEvoy et al., 1998; Monfort & Pouthas, 2003). Thus, while the LPP may primarily reflect the affective salience of imagery or imagery cues, the frontocentral negativity observed in the present study may index the cognitive effort involved in generating and maintaining imagery. Future studies with greater statistical power are needed to test whether the tentative difference between aversive and neutral imagery cues is robust and clarify its functional relevance, specifically, whether this frontocentral negativity is sensitive to affective content or whether electric shocks are simply more difficult to imagine than vibrations.

In addition to the LPP as a new outcome measure in the context of imagery-based fear learning, we largely replicated previous findings of our imagery-based fear conditioning paradigm, namely for subjective ratings, SCR, and evoked IBI. As in our previous study (Mueller et al., 2019), participants reported higher fear ratings to aversive CS+ compared with both neutral CS+ and CS−. This was also true for unpleasantness and arousal ratings (reported in Supplement 1), with the single exception of a non-significant difference between aversive CS+ and neutral CS+ in arousal ratings. In contrast to ratings, we found no significant effects of CS type for the peripheral autonomic measures in the imagery-based conditioning group of the current sample. This lack of differential SCR conditioning is in line with our two previous samples (Mueller et al., 2019) and further corroborates the assumption that electrodermal activity is not sensitive to imagery-based fear conditioning—most likely due to the absence of an external event as US, as elaborated below. Meanwhile, the IBI in the a priori time window (i.e., 2 to 5 s post-CS) descriptively showed comparable response patterns to previous samples as well as relative cardiac slowing to CS+_av_. However, the effect of CS Type did not reach significance in the current study, which is in contrast to these samples (Mueller et al., 2019). Given that previous findings on IBI were somewhat less consistent across samples than ratings and given that the current imagery-based conditioning group was considerably smaller than the sample of our previous study, it appears that effects of imagery-based fear conditioning on evoked IBI are more subtle and thus require larger samples to be reliably detected than other outcome measures. This may be, in part, due to multiple simultaneously active sub-processes contributing to net changes in IBI. Imagery itself is typically accompanied by cardiac acceleration (Cuthbert et al., 2003; Vrana & Lang, 1990) as external sensory information is rejected. Along these lines, we have previously argued that imagery-based conditioning may induce an acceleratory component that is superimposed on decelerative components related to threat-anticipation and that is absent in classical fear conditioning. This interpretation is also supported by the present findings of general cardiac acceleration in the imagery-based compared with the classical fear conditioning group.

In order to directly compare conditioned responses from imagery-based conditioning with classical conditioning, the current study also included an experimental group in which CS predicted physical shock and vibration US. Notably, both experimental groups together showed increased LPP amplitudes to aversive CS+ and differential conditioning effects did not differ significantly between groups. This suggests that CS paired with aversive imagery acquire similar motivational significance and evoke comparable neural evaluation processes as classically conditioned CS+. Moreover, not only does CS evaluation appear to be comparable on the neural level, but also on the explicit, verbal level given the comparable differential fear ratings between the conditioning groups. In contrast to LPP and ratings, peripheral autonomic measures (i.e., SCR and IBI) significantly differed between the experimental groups. For IBI in classical conditioning, we found strong cardiac deceleration to CS+_av_, both compared with CS+_neu_ and CS−, in the exploratory time window immediately before US onset. This effect, however, was absent in imagery-based conditioning. Cardiac deceleration in the affective-motivational context has been suggested to reflect increased sensory intake and attentional orienting (Bradley, 2009; Graham & Clifton, 1966; Vila et al., 2007). Moreover, late cardiac deceleration has repeatedly been observed in paradigms in which a cue predicts a second, motivationally relevant stimulus (Koers et al., 1997; Löw et al., 2015; Otten et al., 1995; Wendt et al., 2017), with stronger responses during the anticipation of more arousing stimuli (Poli et al., 2007). We therefore argue that the magnitude of late cardiac deceleration in fear conditioning may primarily depend on US characteristics and is more pronounced when expecting physical electrical shocks compared with imagery thereof. Although it is possible that there is a relative deceleration in response to aversive CS+ in imagery-based conditioning (Mueller et al., 2019), contributions of CS processing to the cardiac response appear to be less pronounced than those of anticipating a physical US.

Paralleling IBI analyses, only the classical conditioning group showed SCR potentiation to aversive CS+, which has been interpreted to reflect awareness of CS-US contingencies (Hamm & Vaitl, 1996). Moreover, SCRs to cue stimuli require the anticipation of an actual external stimulus that is motivationally relevant as opposed, for example, to anticipating a behavioral go signal, which is also relevant but not external (Löw et al., 2015; Wendt et al., 2017). The interpretation that differences between imagery-based and classical conditioning are qualitative rather than quantitative is supported by the observation that CS+_av_ in imagery-based conditioning elicited fewer detectable SCRs than in classical conditioning but produced a comparable number of SCRs as all other CS (including CS−; see Supplement 2). In other words, a face CS predicting aversive imagery had the same likelihood of evoking an SCR as a CS predicting the absence of a physical or imagined US. This pattern aligns with the notion that autonomic arousal in threat contexts is tightly linked to preparing for fight‑or‑flight behavior in response to imminent external threat (Fanselow, 2018; Hashemi et al., 2019; Löw et al., 2015). By contrast, internally generated threat does not require mobilization of autonomic resources for behavioral adaptation, resulting in the absence of measurable SCRs and reduced cardiac responses. Taken together, imagery-based conditioning appears to induce comparable conditioned responses related to the processing of the CS motivational relevance, both on the neural (LPP) and subjective level (fear ratings). Meanwhile, autonomic responses that are associated US anticipation and/or behavioral adaptation (e.g., SCR, late IBI) may be completely absent when using imagery as US.

Our current and previous findings suggest a potential role of imagery-based conditioning in the development and maintenance of stable fears without prior experiences of physical aversive events. In general, three different pathways of fear acquisition have been suggested: conditioning, instructed learning, and observational/vicarious learning (Cameron et al., 2016; Rachman, 1977; but also see Poulton & Menzies, 2002). In many cases, when no relevant aversive real-life experiences can be identified, it may appear plausible to automatically attribute the development and maintenance of fears to mechanisms of instructed (Field & Lawson, 2003; Krypotos et al., 2020) or observational learning (Askew & Field, 2007; 2008; Olsson & Phelps, 2004; 2007). Based on the current and previous results (Mueller et al., 2019), however, we suggest that conditioning mechanisms still can contribute to these fears, mediated via aversive imagery US. Moreover, the different mechanisms are not mutually exclusive and the development of fears may often be mediated via more than one pathway (Ollendick & King, 1991). At this point, questions about the ecological relevance of imagery-based fear conditioning relative to other mechanisms remain and easy answers are unlikely given that the contributions of different learning mechanism (i.e., conditioned, instructed, observational) to acquired fears vary between samples and specific fears (Muris et al., 1997; Ollendick & King, 1991; Öst & Hugdahl, 1983). Given that aversive imagery can also be used to strengthen a CS-US association previously established with physical US (Jones & Davey, 1990; Joos et al., 2012), we suggest that imagery-based fear conditioning may be particularly effective in consolidating fears via repeated pairing of CS and (mental) US, regardless of how the fear was acquired in the first place. Even with relatively weak or nonexisting conditioned autonomic responses, aversive imagery appears to have a similar impact on affective evaluation of preceding CS (indexed by LPP and fear ratings) as aversive physical experiences. Imagery-based conditioning, in turn, may be sufficient to promote dysfunctional avoidance of CS (Krypotos et al., 2020), which prevents corrective experiences (i.e., extinction) and thereby contributes even further to temporally stable fears (Pittig et al., 2020; Wong et al., 2022). Moreover, imagery processes might lead to fear generalization (Lyons et al., 2024; van Dis et al., 2024), further contributing to trans-situationally stable fears.

As noted earlier, the current findings may also have implications for clinical research and practice, particularly in the context of phobias and other anxiety disorders. Similar to nonpathological fears, threat appraisals in clinical populations may be acquired and maintained through imagery-based learning processes, as reflected in increased LPP amplitudes and verbal reports. The absence of autonomic fear responses, however, may result in lower perceived distress and less impact on well-being (Miloyan & Pachana, 2015; Wilmer et al., 2021). In cases without aversive first-hand encounters with the feared object, the individuals’ symptoms may not reach thresholds for clinical relevance. On the other hand, the lack of autonomic responses might also lead to overlooking the role of repeated aversive imagery in consolidating threat associations, especially associations that previously have evoked autonomic responses during physical encounters (Arntz et al., 1997; Davey & Matchett, 1994; Jones & Davey, 1990; Yaremko & Werner, 1974). Here, psychoeducation on the effects of aversive imagery, along with guidance on how to manage it, may be useful elements in therapy. Importantly, rather than avoiding imagery altogether, patients may use imagery constructively to reduce fear (Blackwell, 2021; Mitra & Asthana, 2024 for reviews). To this end, for example, in-sensu exposure (Dadds et al., 1997; Hoppe et al., 2022; Jiang & Greening, 2021), imagery rescripting (Dibbets et al., 2012; 2018; Kunze et al., 2019), or positive imagery training (Zbozinek et al., 2015) may be conducted, making imagery a valuable tool in clinical practice. Future studies may use central neural markers, such as the LPP to better understand mechanisms underlying imagery-based development and consolidation maintenance of anxiety disorders as well as imagery-based therapeutic interventions.

The current paradigm has limitations that warrant some caution when inferring the mechanisms underlying our effects. First, trial durations were different between experimental groups due to different durations of the imagery and the physical US. Shock US of 0.5 s duration are very common in fear conditioning research (Sperl et al., 2016), and significantly longer stimulation would be ethically problematic. Meanwhile, in two previous samples (Mueller et al., 2019), we presented imagery cues for 1 or for 3 s, respectively, and found that participants were more successful generating vivid imagery in the longer time window. In order to obtain potent but ethically acceptable imagery/US while also having identical CS-US intervals, different durations for imagery cues and physical stimulation, as well as for CS were chosen. Second, with 24 participants per conditioning group, the study may provide limited statistical power to detect modest effects. This limitation is most relevant for LPP analyses, where some of the a-priori pairwise comparisons only approached conventional significance thresholds. Replication with larger samples will increase confidence in these particular findings. In contrast, ratings and peripheral responses yielded clear patterns with differences qualified by relatively large effect sizes. Evidence for these patterns can be considered robust given their consistency with previous work, both in imagery‑based and classical fear conditioning. Third, we did not screen participants for prior experience with electro‑tactile stimulation and therefore cannot rule out that variations in familiarity might have influenced the vividness or details of the imagined shock. It is worth noting, however, that the provided imagery scripts were very detailed and described intense experiences, thereby facilitating vivid mental imagery even in naïve participants and limiting individual differences in the imagery aversiveness to some degree. Fourth, we cannot entirely rule out second-order conditioning (Jara et al., 2006; Rizley & Rescorla, 1972) as a relevant mechanism in our paradigm. Here, the conditioned responses may have been caused by the CS being paired with a cue that previously had acquired aversive properties itself by being explicitly associated with aversive imagery during the instruction phase—rather than being a consequence of a direct CS-imagery association. Regardless of whether the association is direct or mediated, the present findings show that the cueing of aversive imagery is sufficient for new fear learning. Moreover, if second-order conditioning is relevant for imagery-based conditioning in the laboratory, this likely would transfer to real-life fears as well. For example, the sight of a dog (imagery cue) might elicit aversive imagery of being attacked (US). Over time then, the public park (CS) may become associated with the presence of dogs and acquire threat characteristics.

## Conclusion

The present study showed that imagery-based fear conditioning evokes increased amplitudes of the LPP, a neural marker for facilitated perceptual and attentional processing in affective stimuli. While anticipatory defensive responses (such as IBI and SCR) appear to be mostly exclusive to classical fear conditioning, CS evaluation processes (such as LPP and fear ratings) might be comparable between imagery-based conditioning and conditioning using physical US. Based on these findings, we argue that inherently safe stimuli that are repeatedly followed by aversive imagery may acquire threat characteristics on the central neural level and thereby contribute to the development and maintenance of stable fears.

## Supplementary Information

Below is the link to the electronic supplementary material.

Supplementary file1 (PDF 1639 KB)

## Data Availability

We provide the Presentation code for the stimulus presentation (with exception of the pictures from the Ekman series due to copyright), as well as preprocessed data on the Open Science Framework (https://osf.io/f36jb). Raw physiological data are available upon request.

## Code availability

We provide R scripts for statistical analyses on the Open Science Framework (https://osf.io/f36jb).

## Declarations

### Open practices statement

We provide the Presentation code for the stimulus presentation (with exception of the pictures from the Ekman series due to copyright), preprocessed data, as well as R scripts for statistical analyses and plotting on the Open Science Framework (https://osf.io/f36jb). Raw physiological data are available upon request. This study was preregistered on OSF (https://osf.io/sygd6). This manuscript was made available as a preprint on PsyArXiv (https://osf.io/preprints/psyarxiv/y3nm5).
