# Supplementary material for: Imagery-based fear conditioning enhances the late positive potential
Source: Cogn Affect Behav Neurosci. 2026 Jan 8;26(3):1081–100. doi: 10.3758/s13415-025-01385-y (PMC13260277; doi:10.3758/s13415-025-01385-y)
Supplement: Supplementary file 1 — Supplementary file1 (PDF 1639 KB) [file 13415_2025_1385_MOESM1_ESM.pdf]

Supplement

Supplement 1 – Main Analyses for Unpleasantness, Arousal, Anger, & Disgust Ratings

Summary

Identical to the main analyses on fear ratings, we conducted one-factorial ANOVAs (factor: CS Type) and follow-up t-tests for unpleasantness, arousal, anger, and disgust ratings as dependent variables. Across, the different ratings, we found general conditioning effects in both the imagery-based and classical conditioning groups.

More specifically, in both groups, $CS+_{av}$ evoked higher unpleasantness, anger, and disgust ratings than $CS+_{neu}$ and CS- while $CS+_{neu}$ and CS- did not differ significantly. In addition, there was a Conditioning Group x CS Type interaction for unpleasantness ratings. Follow-up t-tests revealed that participants in the classical conditioning group differentiated more strongly between $CS+_{av}$ and $CS+_{neu}$ compared to participants in the imagery-based conditioning group. Finally, there was a two-way interaction for arousal ratings. In the imagery-based conditioning group, both $CS+_{av}$ and $CS+_{neu}$ were rated significantly higher than CS- but did not differ significantly from one another. Meanwhile, in the classical conditioning group, there were significant differences between all CS ($CS+_{av}$ > $CS+_{neu}$ > CS-). No significant interactions emerged for anger and disgust ratings. Results are also shown in Figure S1.

Results

Group comparison. For unpleasantness ratings, the Conditioning Group x CS Type ANOVA revealed a significant main effect for CS Type ($F(2, 92) = 41.52$, $p_{GG} < .001$, $\eta_p^2 = .474$, $BF_{Incl} = 5.28$ e+13) with higher unpleasantness ratings for $CS+_{av}$ vs. $CS+_{neu}$ ($t(47) = 6.96$, $p_{one\text{-}tailed} < .001$, $d = 1.00$, $BF_{10,\ one\text{-}tailed}$ = 2.65 e+6) and for $CS+_{av}$ vs. CS- ($t(47) = 7.02$, $p_{one\text{-}tailed} < .001$, $d = 1.01$, $BF_{10,\ one\text{-}tailed}$ = 3.24 e+6) but no difference between $CS+_{neu}$ and CS- ($t(47) = 1.37$, $p_{two\text{-}tailed} = .176$, $d = 0.20$, $BF_{10,\ two\text{-}tailed} = 0.38$). Meanwhile, there was no significant main effect of Conditioning Group ($F(2, 46) = 0.73$, $p = .399$, $\eta_p^2 = .002$, $BF_{Incl} = 0.26$). The Conditioning Group x CS Type interaction, however, was significant ($F(2, 92) = 7.10$, $p_{GG} = .018$, $\eta_p^2 = .089$, $BF_{Incl} = 7.24$). This was driven by the pattern that the classical conditioning group showed stronger differential responses for $CS+_{av}$ compared to $CS+_{neu}$ ($t(46) = 2.98$, $p_{Bonferroi} = .014$, $d = 0.86$, $BF_{10,\ two\text{-}tailed} = 9.1$). Meanwhile, there was no evidence for differences between groups for $CS+_{av}$ compared to CS- ($t(46) = 1.87$, $p_{Bonferroni} = .202$, $d = 0.54$, $BF_{10,\ two\text{-}tailed} = 1.2$), or for $CS+_{neu}$ compared to CS- ($t(46) = -0.82$, $p_{Bonferroni} = 1$, $d = -0.24$, $BF_{10,\ wo\text{-}tailed} = 0.38$).

ANOVA on arousal ratings again revealed a significant main effect for CS Type ($F(2, 92) = 38.67$, $p < .001$, $\eta_p^2 = .457$, $BF_{Incl} = 3.71$ e+10) with higher arousal ratings for $CS+_{av}$ vs. $CS+_{neu}$ ($t(47) = 4.02$, $p_{one\text{-}tailed} < .001$, $d = 0.58$, $BF_{10,\ one\text{-}tailed} = 234.97$), for $CS+_{av}$ vs. CS- ($t(47) = 7.52$, $p_{one\text{-}tailed} < .001$, $d = 1.09$, $BF_{10,\ one\text{-}tailed} = 1.73$ e+7), and for $CS+_{neu}$ vs. CS- ($t(47) = 4.71$, $p_{two\text{-}tailed} < .001$, $d = 0.68$, $BF_{10,\ two\text{-}tailed} = 916.09$). There was no significant main effect of Conditioning Group ($F(2, 46) = 3.33$, $p = .075$, $\eta_p^2 = .067$, $BF_{Incl} = 1.00$). Meanwhile, the Conditioning Group x CS Type interaction was significant ($F(2, 92) = 8.41$, $p < .001$, $\eta_p^2 = .155$, $BF_{Incl} = 73.25$). Looking at pairwise comparisons, differential responses for $CS+_{av}$ compared to $CS+_{neu}$ were larger in the classical conditioning group than the (non-significant) difference in the imagery-based conditioning group ($t(46) = 3.35$, $p_{Bonferroi} = .005$, $d = 0.97$, $BF_{10,\ two\text{-}tailed} = 21.02$). Moreover, the classical conditioning group showed stronger differentiation between $CS+_{av}$ and CS- ($t(46) = 3.42$, $p_{Bonferroni} = .004$, $d = 0.99$, $BF_{10,\ two\text{-}tailed} = 24.71$) while the lack of differences between $CS+_{neu}$ and CS- was comparable in both groups ($t(46) = 0.13$, $p_{Bonferroni} = 1$, $d = 0.04$, $BF_{10,\ two\text{-}tailed} = 0.29$).

For anger ratings we found a significant main effect for CS Type ($F(2, 92) = 42.16$, $p_{GG} < .001$, $\eta_p^2 = .478$, $BF_{Incl} = 7.93$ e+11) with higher anger ratings for $CS+_{av}$ vs. $CS+_{neu}$ ($t(47) = 7.15$, $p_{one\text{-}tailed} < .001$, $d = 1.03$, $BF_{10,\ one\text{-}tailed} = 4.99$ e+6) and for $CS+_{av}$ vs. CS- ($t(47) = 7.12$, $p_{one\text{-}tailed} < .001$, $d = 1.03$, $BF_{10,\ one\text{-}tailed} = 4.50$ e+6) but no difference between $CS+_{neu}$ and CS- ($t(47) = 1.60$, $p_{two\text{-}tailed} = .117$, $d = 0.23$, $BF_{10,\ two\text{-}tailed} = 0.51$). Meanwhile, there was no evidence for the main effect of Conditioning Group

($F(2, 46) = 0.11$, $p = .740$, $\eta_p^2 = .002$, $BF_{Incl} = 0.26$) or for the Conditioning Group x CS Type interaction ($F(2, 92) = 1.81$, $p_{GG} = .178$, $\eta_p^2 = .155$, $BF_{Incl} = 0.49$).

Last, for disgust ratings we found a significant main effect for CS Type ($F(2, 92) = 18.30$, $p_{GG} < .001$, $\eta_p^2 = .285$, $BF_{Incl} = 143{,}760$) with higher disgust ratings for $CS+_{av}$ vs. $CS+_{neu}$ ($t(47) = 4.92$, $p_{one\text{-}tailed} < .001$, $d = 0.71$, $BF_{10,\ one\text{-}tailed} = 3{,}556$) and for $CS+_{av}$ vs. CS- ($t(47) = 4.85$, $p_{one\text{-}tailed} < .001$, $d = 0.70$, $BF_{10,\ one\text{-}tailed} = 2{,}821$) but no difference between $CS+_{neu}$ and CS- ($t(47) = 1.13$, $p_{two\text{-}tailed} = .262$, $d = 0.16$, $BF_{10,\ two\text{-}tailed} = 0.29$). Again, there was no evidence for the main effect of Conditioning Group ($F(2, 46) = 1.83$, $p = .183$, $\eta_p^2 = .038$, $BF_{Incl} = 0.55$) or for the Conditioning Group x CS Type interaction ($F(2, 92) = 0.33$, $p_{GG} = .686$, $\eta_p^2 = .007$, $BF_{Incl} = 0.15$).

Imagery-based conditioning group. In the imagery-based conditioning group, unpleasantness ratings showed a significant main effect of CS Type ($F(2, 46) = 10.44$, $p < .001$, $\eta_p^2 = .312$, $BF_{10} = 1{,}007$). The main effect was qualified by higher unpleasantness ratings for $CS+_{av}$ compared to $CS+_{neu}$ ($t(23) = 3.52$, $p_{one\text{-}tailed} < .001$, $d = 0.72$, $BF_{10,\ one\text{-}tailed} = 41.0$) and $CS+_{av}$ compared to CS- ($t(23) = 3.79$, $p_{one\text{-}tailed} < .001$, $d = 0.77$, $BF_{10,\ one\text{-}tailed} = 73.6$). Again, there was no significant difference between $CS+_{neu}$ and CS- ($t(23) = 1.45$, $p_{two\text{-}tailed} = .161$, $d = 0.30$, $BF_{10,\ two\text{-}tailed} = 0.54$).

Although the ANOVA on arousal ratings showed a significant main effect of CS Type as well ($F(2, 46) = 8.14$, $p < .001$, $\eta_p^2 = .261$, $BF_{10} = 41.8$), the pattern differed from the other two ratings. As predicted, the $CS+_{av}$ was rated more arousing than the CS- ($t(23) = 3.70$, $p_{one\text{-}tailed} < .001$, $d = 0.76$, $BF_{10,\ one\text{-}tailed} = 61.0$) although it was not rated more arousing than $CS+_{neu}$ ($t(23) = 0.74$, $p_{one\text{-}tailed} = .23$, $d = 0.15$, $BF_{10,\ one\text{-}tailed} = 0.42$) and a two-tailed t-test revealed higher arousal ratings to $CS+_{neu}$ compared to CS- ($t(23) = 3.71$, $p_{two\text{-}tailed} = .001$, $d = 0.76$, $BF_{10,\ two\text{-}tailed} = 31.3$).

Anger ratings showed a significant main effect of CS Type ($F(2, 46) = 15.18$, $p < .001$, $\eta_p^2 = .398$, $BF_{10} = 5{,}629$). The main effect was qualified by higher anger ratings for $CS+_{av}$ compared to $CS+_{neu}$ ($t(23) = 4.44$, $p_{one\text{-}tailed} < .001$, $d = 0.91$, $BF_{10,\ one\text{-}tailed} = 307.7$) and $CS+_{av}$ compared to CS- ($t(23) = 4.38$, $p_{one\text{-}tailed} < .001$, $d = 0.89$, $BF_{10,\ one\text{-}tailed} = 267.2$). Again, there was no significant difference between $CS+_{neu}$ and CS- ($t(23) = 1.79$, $p_{two\text{-}tailed} = .086$, $d = 0.37$, $BF_{10,\ two\text{-}tailed} = 0.85$).

Last, disgust ratings also showed a significant main effect of CS Type ($F(2, 46) = 10.76$, $p < .001$, $\eta_p^2 = .319$, $BF_{10} = 235.7$). This effect was qualified by higher disgust ratings for $CS+_{av}$ compared to $CS+_{neu}$ ($t(23) = 3.89$, $p_{one\text{-}tailed} < .001$, $d = 0.79$, $BF_{10,\ one\text{-}tailed} = 91.4$) and $CS+_{av}$ compared to CS- ($t(23) = 4.05$, $p_{one\text{-}tailed} < .001$, $d = 0.83$, $BF_{10,\ one\text{-}tailed} = 129.3$). There was no significant difference between $CS+_{neu}$ and CS- ($t(23) = 1.13$, $p_{two\text{-}tailed} = .271$, $d = 0.23$, $BF_{10,\ two\text{-}tailed} = 0.38$).

Classical conditioning group. In the classical conditioning group, unpleasantness ratings yielded a comparable main effect of CS Type ($F(2, 46) = 35.02$, $p < .001$, $\eta_p^2 = .604$, $BF = 5.91$ e+9). This main effect was qualified by higher unpleasantness ratings for $CS+_{av}$ compared to $CS+_{neu}$ ($t(23) = 6.87$, $p_{one\text{-}tailed} < .001$, $d = 1.40$, $BF_{one\text{-}tailed} = 64{,}361$) and $CS+_{av}$ compared to CS- ($t(23) = 6.38$, $p_{one\text{-}tailed} < .001$, $d = 1.30$, $BF_{one\text{-}tailed} = 22{,}599$). There was no significant difference between CS+neu and CS- ($t(23) = 0.42$, $p_{two\text{-}tailed} = .679$, $d = 0.09$, $BF_{two\text{-}tailed} = 0.23$).

ANOVA for arousal ratings also showed a significant main effect of CS Type ($F(2, 46) = 36.50$, $p < .001$, $\eta_p^2 = .613$, $BF = 5.31$ e+8). As predicted, the $CS+_{av}$ was rated more arousing than the $CS+_{neu}$ ($t(23) = 5.72$, $p_{one\text{-}tailed} < .001$, $d = 1.17$, $BF_{one\text{-}tailed} = 5{,}307$) and more arousing than the CS- ($t(23) = 7.86$, $p_{one\text{-}tailed} < .001$, $d = 1.60$, $BF_{one\text{-}tailed} = 496{,}429$). In addition, the two-tailed t-test revealed higher arousal ratings to $CS+_{neu}$ compared to CS- ($t(23) = 3.71$, $p_{two\text{-}tailed} = .001$, $d = 0.76$, $BF_{two\text{-}tailed} = 31.3$).

For anger ratings, a significant main effect of CS Type was found ($F(2, 46) = 28.86$, $p < .001$, $\eta_p^2 = .557$, $BF = 1.72$ e+7). The $CS+_{av}$ was rated higher for anger than the $CS+_{neu}$ ($t(23) = 5.86$, $p_{one\text{-}tailed} < .001$, $d = 1.20$, $BF_{one\text{-}tailed} = 7{,}230$) and than the CS- ($t(23) = 5.67$, $p_{one\text{-}tailed} < .001$, $d = 1.16$, $BF_{one\text{-}tailed}$

= 4,830). There was no evidence for different anger ratings between CS+$_{neu}$ and CS- (t(23) = 0.25, $p_{two\text{-}tailed}$ = .802 d = 0.05, $BF_{two\text{-}tailed}$ = 0.22).

Last, ANOVA for disgust ratings also resulted in a significant main effect of CS Type (F(2, 46) = 7.84, p < .001, η_p^2 = .254, BF = 51.23). The CS+$_{av}$ was rated more disgusting than the CS+$_{neu}$ (t(23) = 3.12, $p_{one\text{-}tailed}$ = .002, d = 0.64, $BF_{one\text{-}tailed}$ = 17.99) and more disgusting than the CS- (t(23) = 2.83, $p_{one\text{-}tailed}$ = .005, d = 0.58, $BF_{one\text{-}tailed}$ = 4,830). There was difference in disgust ratings between CS+$_{neu}$ and CS- (t(23) = 0.30, $p_{two\text{-}tailed}$ = .770 d = 0.06, $BF_{two\text{-}tailed}$ = 0.22).

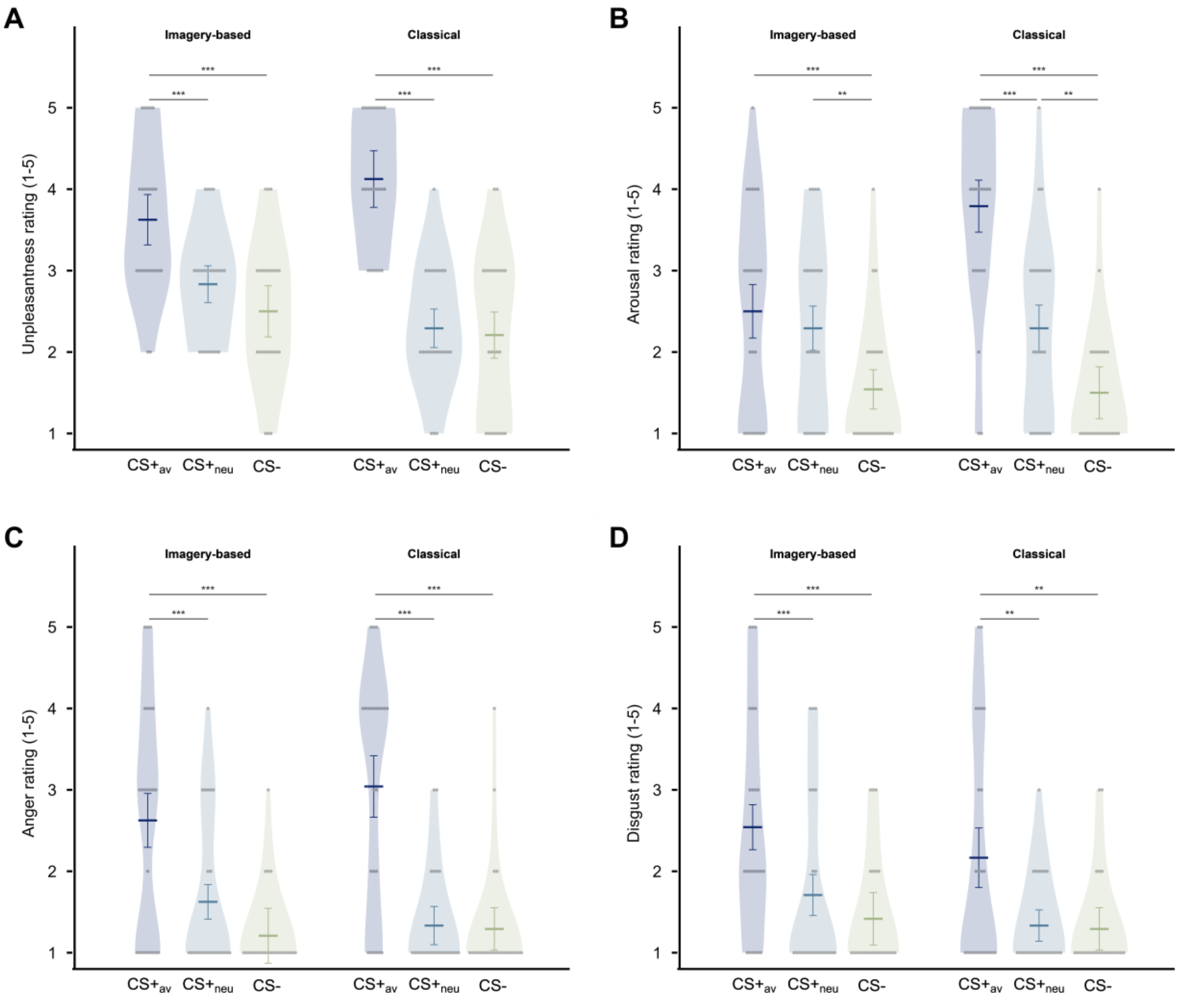

Figure S1. *Individual (dots) and mean (horizontal bars) subjective fear at the end of the experiment for each CS and separate for the imagery-based and the classical conditioning group. Error bars depict the 95% confidence interval of the mean based on within-subject variance. **p < .01, ***p < .001. (A) Unpleasantness ratings (1 = very pleasant, 5 = very unpleasant), (B) Arousal ratings (1 = not arousing, 5 = very arousing), (C) Anger ratings (1 = not angry, 5 = very angry), (D) Disgust ratings (1 = not disgusted, 5 = very disgusted).*

Supplement 2 – Number of non-zero SCRs by Conditioning Group and CS Type

Summary

Main analyses for SCR were repeated with number of non-zero skin conductance responses (> 0.05 µS), using the same parameters for response detection (peak from 1 to 5 s post-CS, baseline-corrected) and the same statistical tests (Tables S1 and S2). Briefly, analyses using the number of non-zero SCRs closely mirrored results from analyses using average SCR amplitude data. There were significantly more responses to $CS+_{av}$ compared to both $CS+_{neu}$ and CS- in the classical conditioning group but not in the imagery-based conditioning group (Figure S2).

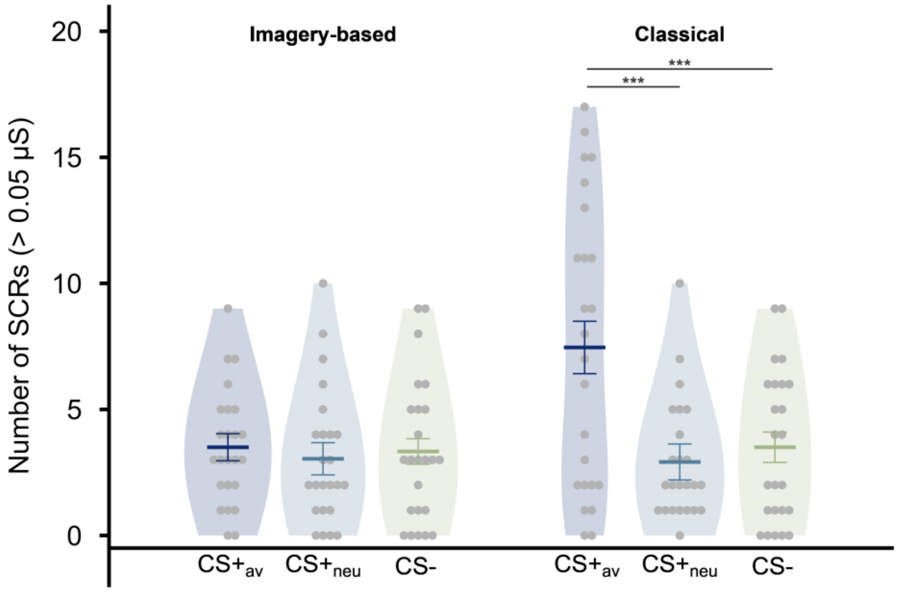

Figure S2. *Individual (dots) and mean (horizontal bars) number of skin conductance responses across trials (> 0.05 µS) for each CS and separate for the imagery-based and the classical conditioning group. Error bars depict the 95% confidence interval of the mean based on within-subject variance. ***p < .001.*

Table S1. ANOVAs on number of non-zero skin conductance responses

	**F**	**Df**	**p**	η_p^2	**BF_{Incl}**
**Across groups**					
Conditioning Group	2.61	1, 46	.113	.054	0.94
CS Type	18.88	2, 92	< .001	.291	92,262
Group x CS Type	13.74	2, 92	< .001	.230	2,763
**Imagery-based**					
CS Type	0.43	2, 46	.650	.019	0.16
**Classical**					
CS Type	24.05	2, 46	< .001	.511	170,216

CS Type x Conditioning Group ANOVA across the two groups. CS Type ANOVAs within the imagery-based conditioning group and within the classical conditioning group. In case of a significant Mauchly test, p-values were corrected using Greenhouse-Geisser correction.

Table S2. t-tests on number of non-zero skin conductance responses

	**t**	**df**	**p**	**d**	**BF_{10}**
**Across groups**					
$CS+_{av} > CS+_{neu}$	4.28	47	< .001[1]	0.62	511.55[1]
$CS+_{av} > CS-$	3.96	47	< .001[1]	0.57	201.55[1]
$CS+_{neu}$ vs. CS-	-1.33	47	.191[2]	-0.19	0.36[2]
**Imagery-based**					
$CS+_{av} > CS+_{neu}$	0.84	23	.205[1]	0.17	0.46[1]
$CS+_{av} > CS-$	0.40	23	.348[1]	0.08	0.30[1]
$CS+_{neu}$ vs. CS-	-0.56	23	.580[2]	-0.11	0.25[2]
**Classical**					
$CS+_{av} > CS+_{neu}$	5.31	23	< .001[1]	1.08	2,143[1]
$CS+_{av} > CS-$	5.04	23	< .001[1]	1.03	1,166[1]
$CS+_{neu}$ vs. CS-	-1-40	23	.175[2]	-0.29	0.51[2]
**Between groups**					
[$CS+_{av}$ - $CS+_{neu}$]	4.03	46	< .001[2, B]	1.16	115.95[2]
[$CS+_{av}$ - CS-]	4.26	46	< .001[2, B]	1.23	216.52[2]
[$CS+_{neu}$ - CS-]	-0.44	46	1[2, B]	-0.13	0.31[2]

t-tests for pairwise comparisons between CS across conditioning groups, within the imagery-based conditioning group and within the classical conditioning group. Tests were conducted one-sided ($CS+_{av} > CS+_{neu}$; $CS+_{av} > CS-$) or two-sided ($CS+_{neu}$ vs. CS-). Positive t-values in the two-sided tests indicate larger values for $CS+_{neu}$. Between groups: Two-sided t-tests between conditioning groups comparing difference values of CS with Bonferroni-corrected p-values. Degrees of freedom were corrected for all between-subject t-tests using the Welch method. In this table, uncorrected degrees of freedom are reported. [1]one-sided test, [2]two-sided test, [B]Bonferroni-corrected for three comparisons.

Supplement 3 – LPP Main Analyses with Preregistered Selection of Time Window, Channel Pz, and mastoids reference

Summary

After inspection of ERPs and their topographies using preregistered parameters (time window: 300 to 700 ms post-CS; channel Pz; mastoid reference), we repeated ANOVAs and *t*-tests of the LPP main analysis with a time window from 400 to 1000 ms, averaging amplitudes across channels Pz, POz, P1, P2, P3, P4, PO3, and PO4 and with an average reference.. Results showed the same general pattern with the one notable difference that the comparison between $CS+_{av}$ and $CS+_{neu}$ in the imagery-based conditioning group was significant in the exploratory analysis while only approaching significance when using preregistered parameters. Here, in the supplement, results from preregistered analyses are reported. For exploratory analyses, see main article.

Results

Table S3. ANOVAs on LPP amplitudes with preregistered time window and channel Pz

	F	df	p	η_p^2	BF_{Incl}
**Across groups**					
Conditioning Group	0.14	1, 46	.708	.003	0.42
CS Type	4.07	2, 92	.020	.081	1.97
Group x CS Type	1.22	2, 92	.300	.026	0.29
**Imagery-based**					
CS Type	1.98	2, 46	.149	.079	0.51
**Classical**					
CS Type	3.10	2, 46	.055	.119	1.14

CS Type x Conditioning Group ANOVA across the two groups. CS Type ANOVAs within the imagery-based conditioning group and within the classical conditioning group. In case of a significant Mauchly test, p-values were corrected using Greenhouse-Geisser correction.

Table S4. t-tests on LPP amplitudes with preregistered time window and channel Pz

	t	df	p	d	BF_{10}
**Across groups**					
$CS+_{av}$ > $CS+_{neu}$	2.87	47	.003[1]	0.41	11.71[1]
$CS+_{av}$ > CS-	1.78	47	.041[1]	0.26	1.29[1]
$CS+_{neu}$ vs. CS-	-0.94	47	.351[2]	-0.14	0.24[2]
**Imagery-based**					
$CS+_{av}$ > $CS+_{neu}$	1.55	23	.068[1]	0.32	1.13[1]
$CS+_{av}$ > CS-	2.01	23	.028[1]	0.41	2.28[1]
$CS+_{neu}$ vs. CS-	0.44	23	.664[2]	0.09	0.23[2]
**Classical**					
$CS+_{av}$ > $CS+_{neu}$	2.43	23	.012[1]	0.50	4.76[1]
$CS+_{av}$ > CS-	0.76	23	.229[1]	0.15	0.42[1]
$CS+_{neu}$ vs. CS-	-1.90	23	.070[2]	-0.39	1.01[2]
**Between groups**					
[$CS+_{av}$ - $CS+_{neu}$]	1.01	46	.959[2, B]	0.29	0.43[2]
[$CS+_{av}$ - CS-]	-0.53	46	1[2, B]	-0.15	0.32[2]
[$CS+_{neu}$ - CS-]	-1.61	46	.344[2, B]	-0.46	0.82[2]

t-tests for pairwise comparisons between CS across conditioning groups, within the imagery-based conditioning group and within the classical conditioning group. Tests were conducted one-sided ($CS+_{av}$ > $CS+_{neu}$; $CS+_{av}$ > CS-) or two-sided ($CS+_{neu}$ vs. CS-). Positive t-values in the two-sided tests indicate larger values for $CS+_{neu}$. Between groups: Two-sided t-tests between conditioning groups comparing difference values of CS with Bonferroni-corrected p-values. Degrees of freedom were corrected for all between-subject t-tests using the Welch method. In this table, uncorrected degrees of freedom are reported. [1]one-sided test, [2]two-sided test, [B]Bonferroni-corrected for three comparisons.

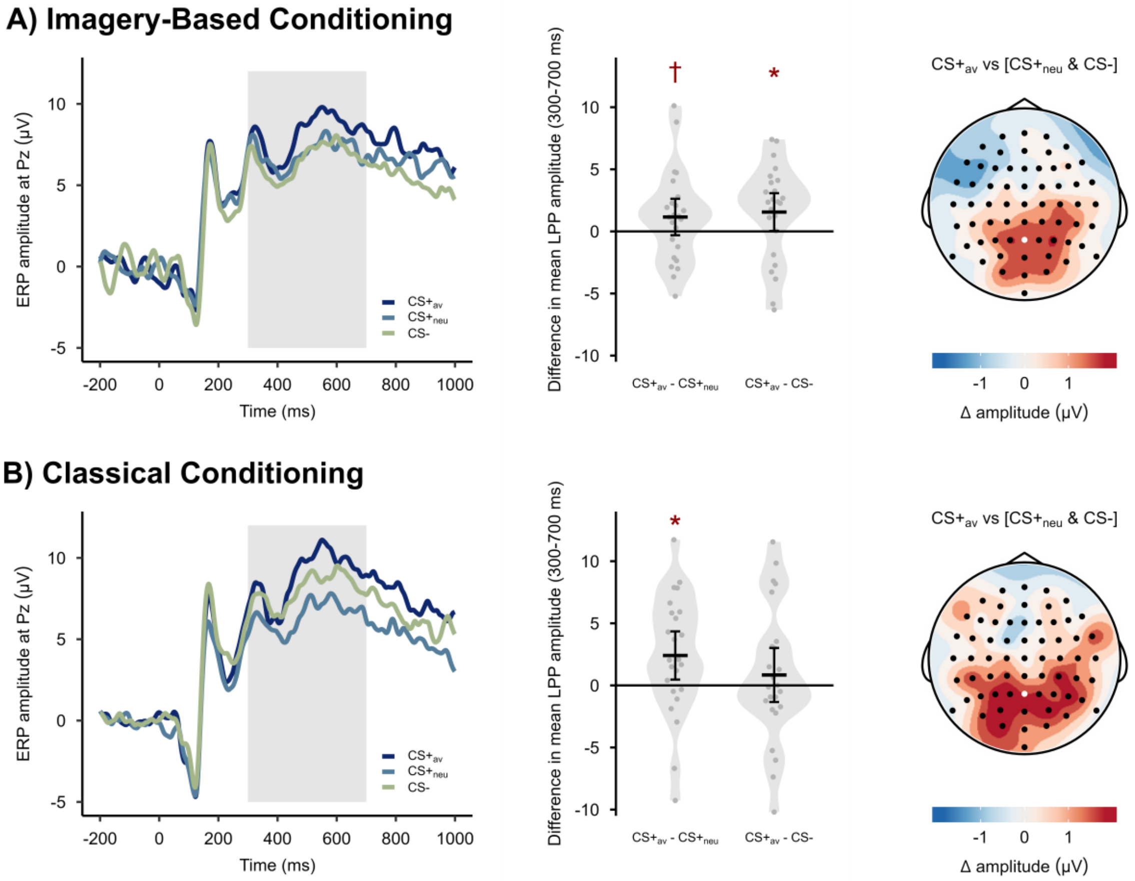

Figure S3. *The Late Positive Potential (LPP) at Pz in response to the different CS and separate for the imagery-based and the classical conditioning group. The shaded areas in the ERP panels indicate the preregistered time window (300 to 700 ms post-CS). The graphs in the middle depict differences of mean LPP amplitudes at Pz from 300 to 700 ms for the comparisons [$CS+_{av}$ – $CS+_{neu}$] and [$CS+_{av}$ – CS-]. Individual values are shown as dots, the group means are shown as horizontal bar. Error bars depict the 95% confidence interval of the mean based on within-subject variance. *$p < .05$, $^{\dagger}p < .10$. Topographies show the contrast comparing $CS+_{av}$ to the other two CS (weights: [1, -0.5, -0.5]).*

Supplement 4 – Main Analyses in Contingency-Aware Participants

Summary

We repeated all main analyses on fear ratings, SCR, IBI, and LPP using only contingency-aware participants. In the imagery-based conditioning group participants completed a pen-and-paper survey after the experiment, in which they were asked to indicate in how many percent of trials they imagined the electric shock/vibration/nothing when seeing each of the CS, respectively. Participants were categorized as contingency-aware (n = 22) if they reported imagining (a) the shock more often after $CS+_{av}$ than after $CS+_{neu}$ or CS-, (b) the vibration more often after $CS+_{neu}$ than after $CS+_{av}$ or CS-, and (c) nothing more often after CS- than after $CS+_{av}$ or $CS+_{neu}$.

In the classical conditioning group participants completed ratings on the screen at the end of the experiment, in which they were asked to indicate on a 4-point Likert scale how often a given CS was followed by the shock or the vibration, respectively (0 = never, 1 = sometimes, 2, = often, 3 = always). Participants were categorized as contingency-aware (n = 22) if they reported experiencing (a) the shock more often after the $CS+_{av}$ compared to $CS+_{neu}$ and CS- and (b) the vibration more often after the $CS+_{neu}$ compared to $CS+_{av}$ and CS-.

Given the high number of contingency-aware participants (22 out of 24 participants in both conditioning groups), the overall result patterns did not change.

Fear Ratings

Table S5. ANOVAs on fear ratings in contingency-aware participants

	F	df	p	η_p^2	BF_{Incl}
**Across groups**					
Conditioning Group	0.02	1, 42	.876	.001	0.25
CS Type	36.42	2, 84	< .001	.464	2.2 e+10
Group x CS Type	2.43	2, 84	.094	.055	0.84
**Imagery-based**					
CS Type	8.69	2, 42	< .001	.293	77.8
**Classical**					
CS Type	35.93	2, 42	< .001	.631	7.0 e+8

CS Type x Conditioning Group ANOVA across the two groups. CS Type ANOVAs within the imagery-based conditioning group and within the classical conditioning group. In case of a significant Mauchly test, p-values were corrected using Greenhouse-Geisser correction.

Table S6. t-tests on fear ratings in contingency-aware participants

	t	df	p	d	BF_{10}
**Across groups**					
$CS+_{av}$ > $CS+_{neu}$	6.59	43	< .001[1]	0.99	5.5 e+5[1]
$CS+_{av}$ > CS-	6.98	43	< .001[1]	1.05	1.9 e+6[1]
$CS+_{neu}$ vs. CS-	0.44	43	.660[2]	0.07	0.17[2]
**Imagery-based**					
$CS+_{av}$ > $CS+_{neu}$	3.18	21	.002[1]	0.68	19.34[1]
$CS+_{av}$ > CS-	3.92	21	< .001[1]	0.84	87.89[1]
$CS+_{neu}$ vs. CS-	0.66	21	.518[2]	0.14	0.27[2]
**Classical**					
$CS+_{av}$ > $CS+_{neu}$	6.74	21	< .001[1]	1.44	31,648[1]
$CS+_{av}$ > CS-	6.11	21	< .001[1]	1.30	8,934[1]
$CS+_{neu}$ vs. CS-	-0.33	21	.747[2]	-0.07	0.23[2]
**Between groups**					
[$CS+_{av}$ - $CS+_{neu}$]	2.04	42	.145[2, B]	0.61	1.52[2]
[$CS+_{av}$ - CS-]	1.41	42	.496[2, B]	0.43	0.66[2]
[$CS+_{neu}$ - CS-]	-0.73	42	1[2, B]	-0.22	0.37[2]

t-tests for pairwise comparisons between CS across conditioning groups, within the imagery-based conditioning group and within the classical conditioning group. Tests were conducted one-sided ($CS+_{av}$ > $CS+_{neu}$; $CS+_{av}$ > CS-) or two-sided ($CS+_{neu}$ vs. CS-). Positive t-values in the two-sided tests indicate larger values for $CS+_{neu}$. Between groups: Two-sided t-tests between conditioning groups comparing difference values of CS with Bonferroni-corrected p-values. Degrees of freedom were corrected for all between-subject t-tests using the Welch method. In this table, uncorrected degrees of freedom are reported. [1]one-sided test, [2]two-sided test, [B]Bonferroni-corrected for three comparisons.

Skin Conductance Responses

Table S7. ANOVAs on skin conductance responses in contingency-aware participants

	F	df	p	η_p^2	BF_{Incl}
**Across groups**					
Conditioning Group	0.42	1, 42	.522	.010	0.30
CS Type	15.82	2, 84	< .001	.274	21,514
Group x CS Type	12.97	2, 84	< .001	.236	2,576
**Imagery-based**					
CS Type	0.15	2, 42	.858	.007	0.14
**Classical**					
CS Type	19.55	2, 42	< .001	.482	78,528

CS Type x Conditioning Group ANOVA across the two groups. CS Type ANOVAs within the imagery-based conditioning group and within the classical conditioning groupIn case of a significant Mauchly test, p-values were corrected using Greenhouse-Geisser correction.

Table S8. t-tests on skin conductance responses in contingency-aware participants

	t	df	p	d	BF_{10}
**Across groups**					
$CS+_{av} > CS+_{neu}$	3.77	43	< .001[1]	0.57	111.85[1]
$CS+_{av} > CS-$	3.87	43	< .001[1]	0.58	144.83[1]
$CS+_{neu}$ vs. CS-	-0.12	43	.907[2]	-0.02	0.16[2]
**Imagery-based**					
$CS+_{av} > CS+_{neu}$	0.37	21	.359[1]	0.08	0.30[1]
$CS+_{av} > CS-$	0.51	21	.307[1]	0.11	0.34[1]
$CS+_{neu}$ vs. CS-	0.20	21	.845[2]	0.04	0.23[2]
**Classical**					
$CS+_{av} > CS+_{neu}$	4.75	21	< .001[1]	1.01	510.25[1]
$CS+_{av} > CS-$	4.90	21	< .001[1]	1.04	702.48[1]
$CS+_{neu}$ vs. CS-	-0.31	21	.756[2]	-0.07	0.23[2]
**Between groups**					
$[CS+_{av} - CS+_{neu}]$	4.07	42	< .001[2, B]	1.23	118.55[2]
$[CS+_{av} - CS-]$	3.95	42	.001[2, B]	1.19	87.44[2]
$[CS+_{neu} - CS-]$	-0.37	42	1[2, B]	-0.11	0.31[2]

t-tests for pairwise comparisons between CS across conditioning groups, within the imagery-based conditioning group and within the classical conditioning group. Tests were conducted one-sided ($CS+_{av} > CS+_{neu}$; $CS+_{av} > CS-$) or two-sided ($CS+_{neu}$ vs. CS-). Positive t-values in the two-sided tests indicate larger values for $CS+_{neu}$. Between groups: Two-sided t-tests between conditioning groups comparing difference values of CS with Bonferroni-corrected p-values. Degrees of freedom were corrected for all between-subject t-tests using the Welch method. In this table, uncorrected degrees of freedom are reported. [1]one-sided test, [2]two-sided test, [B]Bonferroni-corrected for three comparisons.

Interbeat Interval

Table S9. ANOVAs on interbeat interval responses in contingency-aware participants

	F	df	p	η_p^2	BF_{Incl}
**Across groups**					
Conditioning Group	6.79	1, 42	.013	.139	3.18
CS Type	3.32	2, 84	.041	.073	1.33
Group x CS Type	0.47	2, 84	.627	.011	0.18
**Imagery-based**					
CS Type	0.95	2, 42	.394	.043	0.29
**Classical**					
CS Type	2.95	2, 42	.063	.123	1.16

CS Type x Conditioning Group ANOVA across the two groups.CS Type ANOVAs within the imagery-based conditioning group and within the classical conditioning group. In case of a significant Mauchly test, p-values were corrected using Greenhouse-Geisser correction.

Table S10. t-tests on interbeat interval responses in contingency-aware participants

	t	df	p	d	BF_{10}
**Across groups**					
$CS+_{av} > CS+_{neu}$	0.90	43	.187[1]	0.14	0.39[1]
$CS+_{av} > CS-$	2.50	43	.008[1]	0.38	5.14[1]
$CS+_{neu}$ vs. CS-	1.66	43	.104[2]	0.25	0.58s[2]
**Imagery-based**					
$CS+_{av} > CS+_{neu}$	0.96	21	.173[1]	0.21	0.55[1]
$CS+_{av} > CS-$	1.35	21	.096[1]	0.29	0.88[1]
$CS+_{neu}$ vs. CS-	0.45	21	.656[2]	0.10	0.24[2]
**Classical**					
$CS+_{av} > CS+_{neu}$	0.32	21	.377[1]	0.07	0.29[1]
$CS+_{av} > CS-$	2.17	21	.021[1]	0.46	3.02[1]
$CS+_{neu}$ vs. CS-	2.07	21	.051[2]	0.44	1.32[2]
**Between groups**					
$[CS+_{av} - CS+_{neu}]$	-0.43	42	1[2, B]	-0.13	0.32[2]
$[CS+_{av} - CS-]$	0.54	42	1[2, B]	0.16	0.33[2]
$[CS+_{neu} - CS-]$	0.96	40	1[2, B]	0.29	0.43[2]

t-tests for pairwise comparisons between CS across conditioning groups, within the imagery-based conditioning group and within the classical conditioning group. Tests were conducted one-sided ($CS+_{av} > CS+_{neu}$; $CS+_{av} > CS-$) or two-sided ($CS+_{neu}$ vs. CS-). Positive t-values in the two-sided tests indicate larger values for $CS+_{neu}$. Between groups: Two-sided t-tests between conditioning groups comparing difference values of CS with Bonferroni-corrected p-values. Degrees of freedom were corrected for all between-subject t-tests using the Welch method. In this table, uncorrected degrees of freedom are reported. [1]one-sided test, [2]two-sided test, [B]Bonferroni-corrected for three comparisons.

Late Positive Potential

Table S11. ANOVAs on Late Positive Potential amplitudes in contingency-aware participants

	F	df	p	η_p^2	BF_{Incl}
**Across groups**					
Conditioning Group	0.13	1, 42	.724	.003	0.43
CS Type	4.55	2, 84	.013	.098	2.89
Group x CS Type	1.69	2, 84	.191	.039	0.43
**Imagery-based**					
CS Type	2.84	2, 42	.069	.119	0.97
**Classical**					
CS Type	3.31	2, 42	.046	.136	1.35

CS Type x Conditioning Group ANOVA across the two groups. CS Type ANOVAs within the imagery-based conditioning group and within the classical conditioning group. In case of a significant Mauchly test, p-values were corrected using Greenhouse-Geisser correction.

Table S12. t-tests on Late Positive Potential amplitudes in contingency-aware participants

	t	df	p	d	BF_{10}
**Across groups**					
$CS+_{av} > CS+_{neu}$	3.06	43	.002[1]	0.46	18.03[1]
$CS+_{av} > CS-$	2.01	43	.025[1]	0.30	1.98[1]
$CS+_{neu}$ vs. CS-	-0.75	43	.458[2]	-0.11	0.21[2]
**Imagery-based**					
$CS+_{av} > CS+_{neu}$	1.58	21	.065[1]	0.34	1.21[1]
$CS+_{av} > CS-$	2.62	21	.008[1]	0.56	6.70[1]
$CS+_{neu}$ vs. CS-	0.75	21	.462[2]	0.16	0.29[2]
**Classical**					
$CS+_{av} > CS+_{neu}$	2.67	21	.007[1]	0.57	7.28[1]
$CS+_{av} > CS-$	0.71	21	.242[1]	0.15	0.42[1]
$CS+_{neu}$ vs. CS-	-2.01	21	.057[2]	-0.43	1.21[2]
**Between groups**					
[$CS+_{av}$ - $CS+_{neu}$]	1.03	42	.926[2, B]	0.31	0.46[2]
[$CS+_{av}$ - CS-]	-0.79	42	1[2, B]	-0.24	0.38[2]
[$CS+_{neu}$ - CS-]	-1.89	42	.195[1, B]	-0.57	1.23[2]

t-tests for pairwise comparisons between CS within the imagery-based conditioning group and the classical conditioning group. Tests were conducted one-sided ($CS+_{av} > CS+_{neu}$; $CS+_{av} > CS-$) or two-sided ($CS+_{neu}$ vs. CS-). Positive t-values in the two-sided tests indicate larger values for $CS+_{neu}$. Two-sided t-tests between conditioning groups comparing difference values of CS with Bonferroni-corrected p-values. Degrees of freedom were corrected for all between-subject t-tests using the Welch method. In this table, uncorrected degrees of freedom are reported. [1]one-sided test, [2]two-sided test, [B]Bonferroni-corrected for three comparisons.

Supplement 5 – Main Analyses with additional Factor *Time*

Summary

We repeated all main analyses on fear ratings, SCR, IBI, and LPP adding the within-subject factor Time to ANOVAs (levels for ratings: before conditioning vs. midway through acquisition vs. after acquisition; levels for physiological measures: 1st half of trials vs. 2nd half of trials). As for the main analyses, frequentist and Bayesian ANOVAs with Inclusion Bayes factors were computed. Here, however, the *generalTestBF* function from the *BayesFactor* package (Rouder et al., 2012) was used and random slopes for the within-subject factors (CS Type and Time) were included in the null model, in addition to the overall intercept, following recent recommendations (van den Bergh et al., 2023). Follow-up t-tests were computed comparing CS within each level of the Time factor. As for the main analyses, comparisons including the $CS+_{av}$ were one-sided ($CS+_{av} > CS+_{neu}$; $CS+_{av} > CS-$) while the comparison between the control CS ($CS+_{neu}$ vs. CS-) was exploratory and two-sided. To investigate differences in differential learning between he imagery-based and the classical conditioning group, between-subject t-tests were computed for each of the three CS comparisons and p-values were Bonferroni-corrected accordingly. Again, this was repeated for each level of the Time factor.

Briefly, there were no significant differences between conditioning groups in fear ratings (Table S11). In both groups, $CS+_{av}$ were rated more fear-inducing than $CS+_{neu}$ and CS-, midway and after conditioning but not before (Figure S3, Table S12).

As in the main analyses, skin conductance responses differentiated between CS only in the classical conditioning group but not in the imagery-based conditioning group (Table S13). In the classical condtionition group, increased skin conductance responses to $CS+_{av}$ were evident in both halves of the experiment (Figure S4, Table S14). In addition, there was a general habituation effect as responses to all CS decreased from the first to the second half in the classical conditioning group (but not in the imagery-based group).

For interbeat interval responses, ANOVA again revealed a significant main effect of CS Type when testing across groups and a main effect of group indicating relatively shorter intervals in the imagery based conditioning group (Table S15). There was a significant main effect of time, with shorter intervals in the second half, but no interactions including the Time factor (see Figure S5 and Table S16 for detailed results).

Finally, for the LPP, a significant Conditioning Group x CS Type x Time interaction emerged (Table S17). More precisely, a CS Type x Time interaction was found in the imagery-based conditioning group as LPP amplitudes differed between CS in the second half but not in the first one (Figure S6, Table S18). This two-way interaction was absent in the classical conditioning group.

Fear Ratings

Table S13. ANOVAs on fear ratings including Time factor

	F	df	p	η_p^2	BF_{Incl}
**Across groups**					
Conditioning Group	0.25	1, 46	.620	.005	0.30
CS Type	27.79	2, 92	< .001	.377	3.4 e+07
Time	2.47	2, 92	.090	.051	0.15
Group x CS	1.08	2, 92	.344	.023	0.32
Group x Time	0.99	2, 92	.374	.021	0.11
CS x Time	15.99	4, 184	< .001	.258	3.8 e+10
Group x CS x Time	1.95	4, 184	.122	.040	0.73
**Imagery-based**					
CS Type	7.66	2, 46	.001	.250	36.62
Time	3.39	2, 46	.042	.129	0.61
CS Type x Time	5.63	4, 92	< .001	.197	308.50
**Classical**					
CS Type	25.65	2, 46	< .001	.527	5.9 e+05
Time	0.19	2, 46	.830	.008	0.09
CS Type x Time	11.33	4, 92	< .001	.330	1.3 e+07

CS Type x Time ANOVAs within the imagery-based conditioning group and within the classical conditioning group. CS Type x Conditioning Group x Time ANOVA across the two groups. In case of a significant Mauchly test, p-values were corrected using Greenhouse-Geisser correction. For the three-way ANOVA, only 10,000 iterations were used for the Bayesian ANOVA due to the higher number of tested models and increased computational demands.

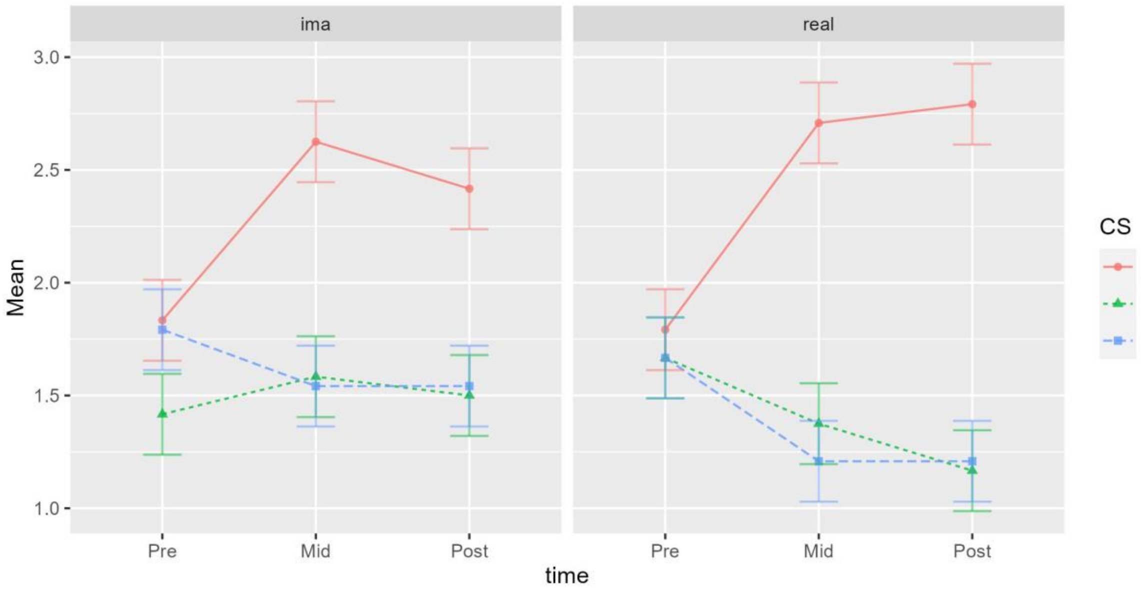

Figure S4. Means and standard errors of the mean for skin conductance responses. *Responses are shown separate for groups (ima: imagery-based conditioning; real: classical conditioning), CS Type (Av: CS+$_{av}$; Neu: CS+$_{neu}$; Min: CS-), and time of rating (Pre: pre-conditioning; Mid: between conditioning blocks; Post: post-conditioning).*

Table S14. t-tests on fear ratings, separate for pre-, mid-, and post-conditioning

		t	df	p	d	BF
**Across groups**						
Pre-conditioning	$CS+_{av}$ vs. $CS+_{neu}$	1.50	47	.140[2]	0.22	0.45[2]
	$CS+_{av}$ vs. CS-	0.46	47	.649[2]	0.07	0.17[2]
	$CS+_{neu}$ vs. CS-	-1.16	47	.253[2]	-0.17	0.29[2]
Mid-conditioning	$CS+_{av}$ > $CS+_{neu}$	6.77	47	< .001[1]	0.98	1.5 e+6[1]
	$CS+_{av}$ > CS-	7.34	47	< .001[1]	1.06	9.3 e+6[1]
	$CS+_{neu}$ vs. CS-	0.71	47	.481[2]	0.10	0.20[2]
Post-conditioning	$CS+_{av}$ > $CS+_{neu}$	7.04	47	< .001[1]	1.02	3.5 e+6[1]
	$CS+_{av}$ > CS-	6.21	47	< .001[1]	0.90	2.2 e+5[1]
	$CS+_{neu}$ vs. CS-	-0.26	47	.796[2]	-0.04	0.16[2]
**Imagery-based**						
Pre-conditioning	$CS+_{av}$ vs. $CS+_{neu}$	1.86	23	.076[2]	0.38	0.94[2]
	$CS+_{av}$ vs. CS-	0.15	23	.880[2]	0.03	0.22[2]
	$CS+_{neu}$ vs. CS-	-1.99	23	.059[2]	-0.41	1.15[2]
Mid-conditioning	$CS+_{av}$ > $CS+_{neu}$	4.40	23	< .001[1]	0.90	280.53[1]
	$CS+_{av}$ > CS-	4.14	23	< .001[1]	0.84	157.51[1]
	$CS+_{neu}$ vs. CS-	0.17	23	.866[2]	0.03	0.22[2]
Post-conditioning	$CS+_{av}$ > $CS+_{neu}$	3.50	23	< .001[1]	0.71	39.62[1]
	$CS+_{av}$ > CS-	2.95	23	.004[1]	0.60	12.69[1]
	$CS+_{neu}$ vs. CS-	-0.14	23	.890[2]	-0.03	0.22[2]
**Classical**						
Pre-conditioning	$CS+_{av}$ vs. $CS+_{neu}$	0.44	23	.664[2]	0.09	0.23[2]
	$CS+_{av}$ vs. CS-	0.51	23	.612[2]	0.10	0.24[2]
	$CS+_{neu}$ vs. CS-	0.00	23	1[2]	0.00	0.21[2]
Mid-conditioning	$CS+_{av}$ > $CS+_{neu}$	5.13	23	< .001[1]	1.05	1,428[1]
	$CS+_{av}$ > CS-	6.43	23	< .001[1]	1.31	2.5 e+04[1]
	$CS+_{neu}$ vs. CS-	1.00	23	.328[2]	0.20	0.34[2]
Post-conditioning	$CS+_{av}$ > $CS+_{neu}$	7.01	23	< .001[1]	1.43	87,662[1]
	$CS+_{av}$ > CS-	6.40	23	< .001[1]	1.31	2.3 e+04[1]
	$CS+_{neu}$ vs. CS-	-0.33	23	.747[2]	-0.07	0.23[2]
**Between groups**						
Pre-conditioning	[$CS+_{av}$ - $CS+_{neu}$]	-0.81	46	1[2, B]	-0.23	0.37[2]
	[$CS+_{av}$ - CS-]	0.23	46	1[2, B]	0.07	0.29[2]
	[$CS+_{neu}$ - CS-]	1.16	46	.757[2, B]	0.34	0.50[2]
Mid-conditioning	[$CS+_{av}$ - $CS+_{neu}$]	0.83	46	1[2, B]	0.24	0.38[2]
	[$CS+_{av}$ - CS-]	1.19	46	.722[2, B]	0.34	0.51[2]
	[$CS+_{neu}$ - CS-]	0.42	46	1[2, B]	0.12	0.31[2]
Post-conditioning	[$CS+_{av}$ - $CS+_{neu}$]	2.03	46	.146[2, B]	0.58	1.48[2]
	[$CS+_{av}$ - CS-]	1.83	46	.221[2, B]	0.53	1.11[2]
	[$CS+_{neu}$ - CS-]	0.00	46	1[2, B]	0.00	0.29[2]

t-tests for pairwise comparisons between CS across conditioning groups, within the imagery-based conditioning group, and the classical conditioning group. Tests were conducted one-sided ($CS+_{av}$ > $CS+_{neu}$ and $CS+_{av}$ > CS- for mid-conditioning and post-conditioning ratings) or two-sided (all comparisons for pre-conditioning ratings; all comparisons $CS+_{neu}$ vs. CS-). Positive t-values in the two-sided tests indicate larger values for $CS+_{neu}$. Two-sided t-tests between conditioning groups comparing difference values of CS with Bonferroni-corrected p-values. Positive t-values indicate larger positive differences in the imagery-based conditioning group. Degrees of freedom were corrected for all between-subject t-tests using the Welch method. In this table, uncorrected degrees of freedom are reported. [1]one-sided test, [2]two-sided test, [B]Bonferroni-corrected for three comparisons.

Skin Conductance Responses

Table S15. ANOVAs on skin conductance responses including Time factor

	F	df	p	η_p^2	BF_{Incl}
**Across groups**					
Conditioning Group	0.26	1, 46	.615	.006	0.26
CS Type	16.53	2, 92	< .001	.264	1.8 e+04
Time	8.91	1, 46	.005	.162	5.92
Group x CS	12.06	2, 92	< .001	.208	778.73
Group x Time	22.45	1, 92	< .001	.328	538.82
CS x Time	0.04	2, 92	.959	.001	0.07
Group x CS x Time	0.96	2, 92	.387	.020	0.29
**Imagery-based**					
CS Type	0.34	2, 46	.717	.014	0.09
Time	2.38	1, 23	.137	.094	0.38
CS Type x Time	0.33	2, 46	.724	.014	0.13
**Classical**					
CS Type	19.27	2, 46	< .001	.456	2.8 e+04
Time	22.04	1, 23	< .001	.489	274.64
CS Type x Time	0.65	2, 46	.526	.028	0.52

CS Type x Conditioning Group x Time ANOVA across the two groups. CS Type x Time ANOVAs within the imagery-based conditioning group and within the classical conditioning group. In case of a significant Mauchly test, p-values were corrected using Greenhouse-Geisser correction. For the three-way ANOVA, only 10,000 iterations were used for the Bayesian ANOVA due to the higher number of tested models and increased computational demands.

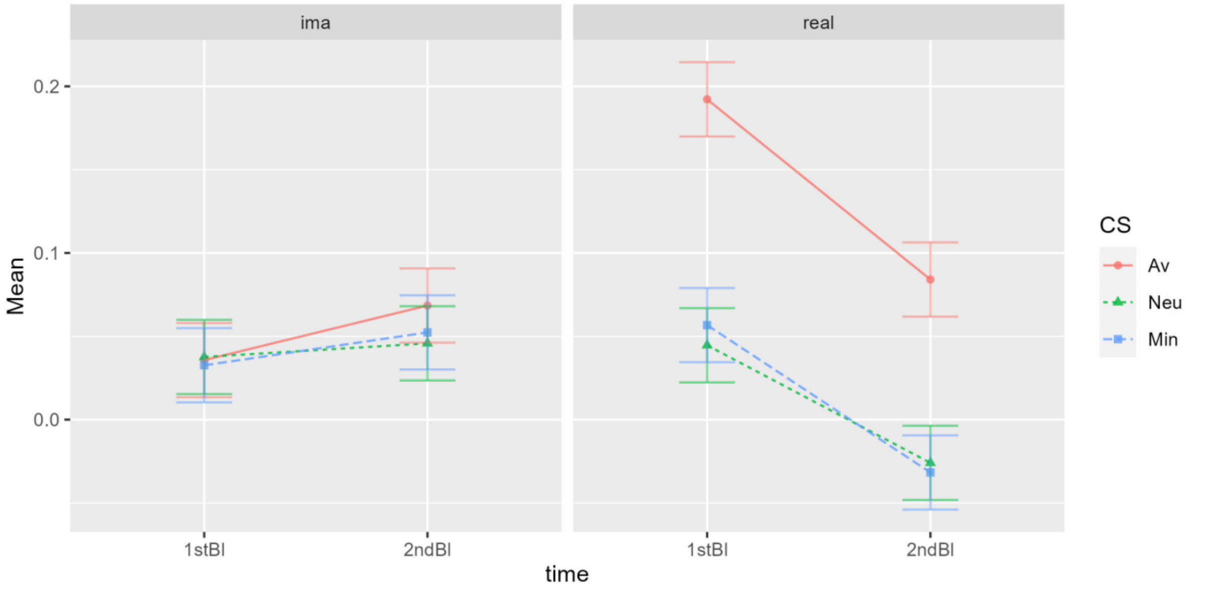

Figure S5. Means and standard errors of the mean for skin conductance responses. *Responses are shown separate for groups (ima: imagery-based conditioning; real: classical conditioning), CS Type (Av: CS+$_{av}$; Neu: CS+$_{neu}$; Min: CS-), and trial block (1stBl: 1st trial block; 2ndBl: 2nd trial block).*

Table S16. t-tests on skin conductance responses, separate for first and and second half of trials

		t	df	p	d	BF_{10}
**Across groups**						
1st trial block	$CS+_{av}$ > $CS+_{neu}$	3.39	47	< .001[1]	0.49	41.74[1]
	$CS+_{av}$ > CS-	3.23	47	.001[1]	0.47	27.54[1]
	$CS+_{neu}$ vs. CS-	-0.20	47	.843[2]	-0.03	0.16[2]
2nd trial block	$CS+_{av}$ > $CS+_{neu}$	3.17	47	.001[1]	0.46	23.79[1]
	$CS+_{av}$ > CS-	3.63	47	< .001[1]	0.52	79.35[1]
	$CS+_{neu}$ vs. CS-	-0.03	47	.974[2]	0.00	0.16[2]
**Imagery-based**						
1st trial block	$CS+_{av}$ > $CS+_{neu}$	-0.13	23	.551[1]	-0.03	0.20[1]
	$CS+_{av}$ > CS-	0.18	23	.430[1]	0.04	0.25[1]
	$CS+_{neu}$ vs. CS-	0.21	23	.834[2]	0.04	0.22[2]
2nd trial block	$CS+_{av}$ > $CS+_{neu}$	0.88	23	.195[1]	0.18	0.48[1]
	$CS+_{av}$ > CS-	0.76	23	.228[1]	0.15	0.42[1]
	$CS+_{neu}$ vs. CS-	-0.33	23	.745[2]	-0.07	0.23[2]
**Classical**						
1st trial block	$CS+_{av}$ > $CS+_{neu}$	4.28	23	< .001[1]	0.87	214.60[1]
	$CS+_{av}$ > CS-	3.90	23	< .001[1]	0.80	94.28[1]
	$CS+_{neu}$ vs. CS-	-0.44	23	.661[2]	-0.09	0.23[2]
2nd trial block	$CS+_{av}$ > $CS+_{neu}$	3.55	23	< .001[1]	0.73	44.35[1]
	$CS+_{av}$ > CS-	4.43	23	< .001[1]	0.90	302.68[1]
	$CS+_{neu}$ vs. CS-	0.40	23	.694[2]	0.08	0.23[2]
**Between groups**						
1st trial block	[$CS+_{av}$ - $CS+_{neu}$]	3.99	46	.001[2, B]	1.15	105.00[2]
	[$CS+_{av}$ - CS-]	3.41	46	.005[2, B]	0.98	24.24[2]
	[$CS+_{neu}$ - CS-]	-0.48	46	1[2, B]	-0.14	0.32[2]
2nd trial block	[$CS+_{av}$ - $CS+_{neu}$]	2.16	46	.108[2, B]	0.62	1.85[2]
	[$CS+_{av}$ - CS-]	2.96	46	.015[2, B]	0.85	8.60[2]
	[$CS+_{neu}$ - CS-]	0.50	46	1[2, B]	0.14	0.32[2]

t-tests for pairwise comparisons between CS across conditioning groups, within the imagery-based conditioning group, and the classical conditioning group. Tests were conducted one-sided ($CS+_{av}$ > $CS+_{neu}$ and $CS+_{av}$ > CS- for mid-conditioning and post-conditioning ratings) or two-sided (all comparisons for pre-conditioning ratings; all comparisons $CS+_{neu}$ vs. CS-). Positive t-values in the two-sided tests indicate larger values for $CS+_{neu}$. Two-sided t-tests between conditioning groups comparing difference values of CS with Bonferroni-corrected p-values. Positive t-values indicate larger positive differences in the imagery-based conditioning group. Degrees of freedom were corrected for all between-subject t-tests using the Welch method. In this table, uncorrected degrees of freedom are reported. [1]one-sided test, [2]two-sided test, [B]Bonferroni-corrected for three comparisons.

Interbeat Interval

Table S17. ANOVAs on interbeat interval responses including Time factor

	**F**	**df**	**p**	η_p^2	BF_{Incl}
**Across groups**					
Conditioning Group	8.16	1, 46	.006	.151	4.80
CS Type	3.80	2, 92	.026	.076	1.01
Time	27.08	1, 46	< .001	.371	538.04
Group x CS	0.24	2, 92	.789	.005	0.11
Group x Time	0.08	1, 46	.781	.002	0.22
CS x Time	1.04	2, 92	.359	.022	0.22
Group x CS x Time	0.14	2, 92	.870	.003	0.14
**Imagery-based**					
CS Type	1.19	2, 46	.314	.049	0.27
Time	12.47	1, 23	.002	.352	5.05
CS Type x Time	0.35	2, 46	.708	.015	0.20
**Classical**					
CS Type	2.91	2, 46	.064	.112	0.75
Time	14.63	1, 23	< .001	.389	8.75
CS Type x Time	0.81	2, 46	.449	.034	0.33

CS Type x Conditioning Group x Time ANOVA across the two groups. CS Type x Time ANOVAs within the imagery-based conditioning group and within the classical conditioning group. In case of a significant Mauchly test, p-values were corrected using Greenhouse-Geisser correction. For the three-way ANOVA, only 10,000 iterations were used for the Bayesian ANOVA due to the higher number of tested models and increased computational demands.

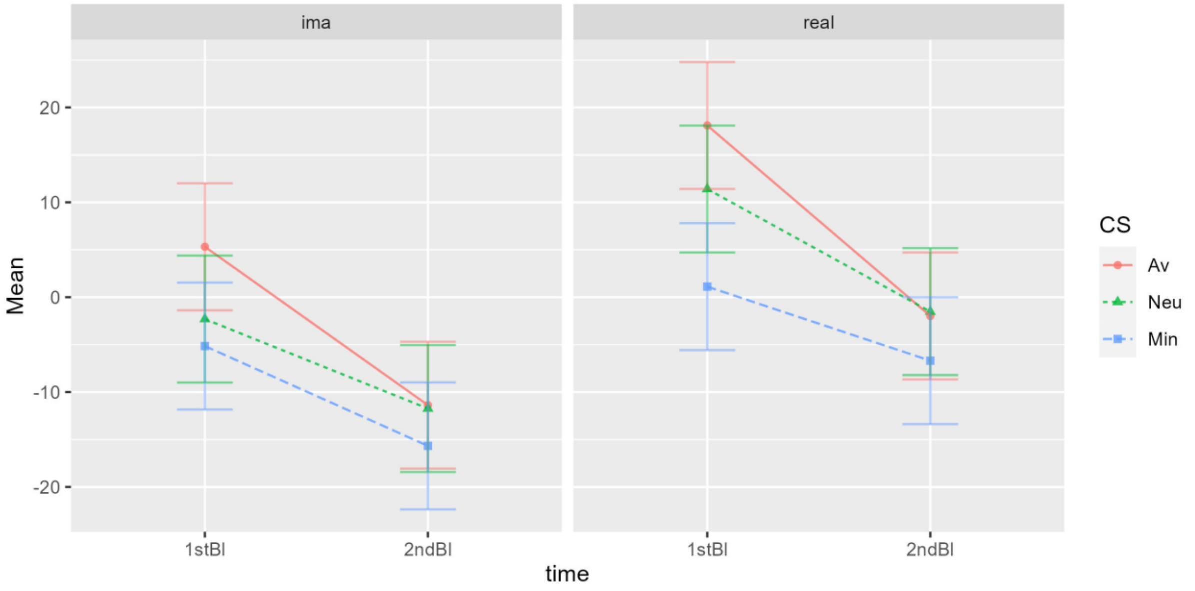

Figure S6. Means and standard errors of the mean for interbeat interval responses. *Responses are shown separate for groups (ima: imagery-based conditioning; real: classical conditioning), CS Type (Av: CS+$_{av}$; Neu: CS+$_{neu}$; Min: CS-), and trial block (1stBl: 1st trial block; 2ndBl: 2nd trial block).*

Table S18. t-tests on interbeat interval responses, separate for first and and second half of trials

		t	df	p	d	BF_{10}
**Across groups**						
1st trial block	$CS+_{av}$ > $CS+_{neu}$	1.60	47	.058[1]	0.23	0.96[1]
	$CS+_{av}$ > CS-	3.23	47	.001[1]	0.47	27.77[1]
	$CS+_{neu}$ vs. CS-	1.74	47	.088[2]	0.25	0.63[2]
2nd trial block	$CS+_{av}$ > $CS+_{neu}$	0.00	47	.504[1]	0.00	0.16[1]
	$CS+_{av}$ > CS-	0.96	47	.170[1]	0.14	0.40[1]
	$CS+_{neu}$ vs. CS-	0.83	47	.411[2]	0.12	0.22[2]
**Imagery-based**						
1st trial block	$CS+_{av}$ > $CS+_{neu}$	1.50	23	.074[1]	0.31	1.05[1]
	$CS+_{av}$ > CS-	2.05	23	.026[1]	0.42	2.44[1]
	$CS+_{neu}$ vs. CS-	0.56	23	.579[2]	0.11	0.25[2]
2nd trial block	$CS+_{av}$ > $CS+_{neu}$	0.04	23	.483[1]	0.01	0.22[1]
	$CS+_{av}$ > CS-	0.57	23	.287[1]	0.12	0.35[1]
	$CS+_{neu}$ vs. CS-	0.49	23	.627[2]	0.10	0.24[2]
**Classical**						
1st trial block	$CS+_{av}$ > $CS+_{neu}$	0.89	23	.190[1]	0.18	0.49[1]
	$CS+_{av}$ > CS-	2.48	23	.010[1]	0.51	5.19[1]
	$CS+_{neu}$ vs. CS-	1.84	23	.079[2]	0.38	0.91[2]
2nd trial block	$CS+_{av}$ > $CS+_{neu}$	0.53	23	.528[1]	-0.01	0.20[1]
	$CS+_{av}$ > CS-	0.21	23	.210[1]	0.17	0.45[1]
	$CS+_{neu}$ vs. CS-	0.51	23	.509[2]	0.14	0.26[2]
**Between groups**						
1st trial block	[$CS+_{av}$ - $CS+_{neu}$]	-0.10	46	1[2, B]	-0.03	0.29[2]
	[$CS+_{av}$ - CS-]	0.76	46	1[2, B]	0.22	0.36[2]
	[$CS+_{neu}$ - CS-]	0.99	46	.986[2, B]	0.28	0.43[2]
2nd trial block	[$CS+_{av}$ - $CS+_{neu}$]	-0.08	46	1[2, B]	-0.02	0.29[2]
	[$CS+_{av}$ - CS-]	0.04	46	1[2, B]	0.01	0.29[2]
	[$CS+_{neu}$ - CS-]	0.11	46	1[2, B]	0.03	0.29[2]

t-tests for pairwise comparisons between CS across conditioning groups, within the imagery-based conditioning group, and the classical conditioning group. Tests were conducted one-sided ($CS+_{av}$ > $CS+_{neu}$ and $CS+_{av}$ > CS- for mid-conditioning and post-conditioning ratings) or two-sided (all comparisons for pre-conditioning ratings; all comparisons $CS+_{neu}$ vs. CS-). Positive t-values in the two-sided tests indicate larger values for $CS+_{neu}$. Two-sided t-tests between conditioning groups comparing difference values of CS with Bonferroni-corrected p-values. Positive t-values indicate larger positive differences in the imagery-based conditioning group. Degrees of freedom were corrected for all between-subject t-tests using the Welch method. In this table, uncorrected degrees of freedom are reported. [1]one-sided test, [2]two-sided test, [B]Bonferroni-corrected for three comparisons.

Late Positive Potential

Table S19. ANOVAs on Late Positive Potential amplitudes including Time factor

	F	df	p	η_p^2	BF_{Incl}
**Across groups**					
Conditioning Group	0.13	1,46	.717	.003	0.41
CS Type	3.66	2,92	.030	.074	0.99
Time	1.65	1,46	.205	.035	0.35
Group x CS	1.11	2,92	.335	.023	0.20
Group x Time	2.98	1,46	.091	.061	0.70
CS x Time	0.23	2,92	.793	.005	0.08
Group x CS x Time	4.25	2,92	.017	.085	5.33
**Imagery-based**					
CS Type	1.787	2,46	.166	.075	0.37
Time	4.65	1,23	.042	.168	1.16
CS Type x Time	3.31	2,46	.046	.126	2.48
**Classical**					
CS Type	2.80	2,46	.071	.108	0.56
Time	0.10	1,23	.760	.004	0.21
CS Type x Time	1.23	2,46	.302	.051	0.31

CS Type x Conditioning Group x Time ANOVA across the two groups. CS Type x Time ANOVAs within the imagery-based conditioning group and within the classical conditioning group. In case of a significant Mauchly test, p-values were corrected using Greenhouse-Geisser correction. For the three-way ANOVA, only 10,000 iterations were used for the Bayesian ANOVA due to the higher number of tested models and increased computational demands.

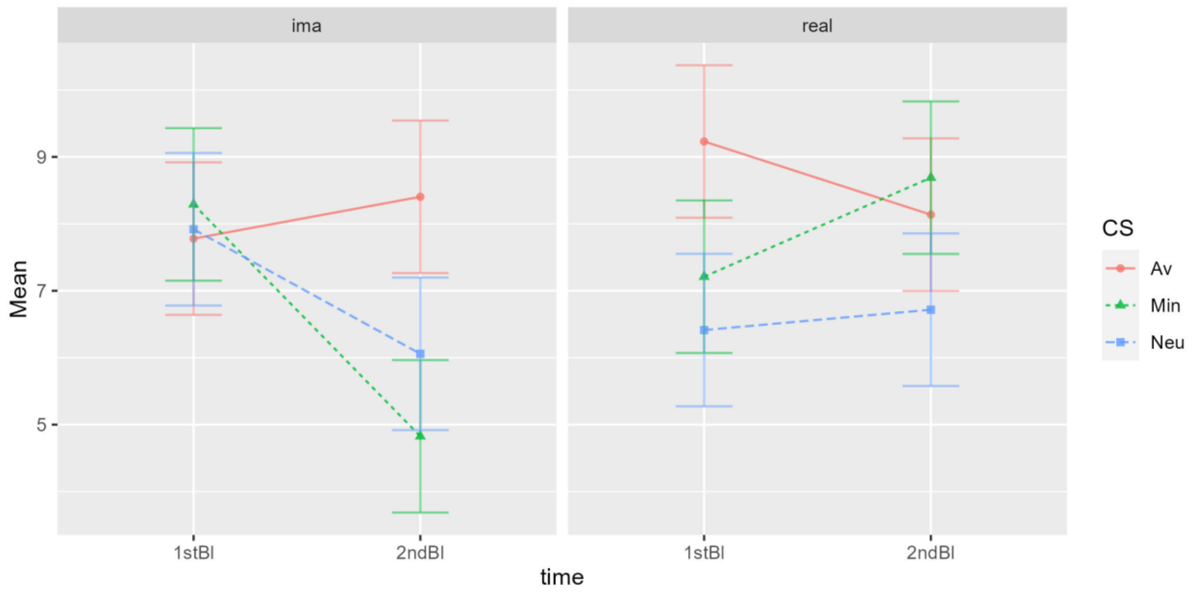

Figure S7. Means and standard errors of the mean for Late Positive Potential amplitudes. *Responses are shown separate for groups (ima: imagery-based conditioning; real: classical conditioning), CS Type (Av: CS+$_{av}$; Neu: CS+$_{neu}$; Min: CS-), and trial block (1stBl: 1st trial block; 2ndBl: 2nd trial block).*

Table S20. t-tests on Late Positive Potential Amplitudes, separate for first and and second half of trials

		t	df	p	d	BF_{10}
**Across groups**						
1st trial block	$CS+_{av} > CS+_{neu}$	1.59	47	.059[1]	0.23	0.95[1]
	$CS+_{av} > CS-$	0.77	47	.222[1]	0.11	0.32[1]
	$CS+_{neu}$ vs. CS-	-0.77	47	.446[2]	-0.11	0.21[2]
2nd trial block	$CS+_{av} > CS+_{neu}$	2.32	47	.012[1]	0.33	3.48[1]
	$CS+_{av} > CS-$	1.74	47	.044[1]	0.25	1.20[1]
	$CS+_{neu}$ vs. CS-	-0.44	47	.661[2]	-0.06	0.17[2]
**Imagery-based**						
1st trial block	$CS+_{av} > CS+_{neu}$	-0.14	23	.556[1]	-0.03	0.19[1]
	$CS+_{av} > CS-$	-0.45	23	.672[1]	-0.19	0.16[1]
	$CS+_{neu}$ vs. CS-	-0.35	23	.729[2]	-0.07	0.23[2]
2nd trial block	$CS+_{av} > CS+_{neu}$	2.18	23	.020[1]	0.44	3.02[1]
	$CS+_{av} > CS-$	2.85	23	.005[1]	0.58	10.41[1]
	$CS+_{neu}$ vs. CS-	0.93	23	.362[2]	0.19	0.32[2]
**Classical**						
1st trial block	$CS+_{av} > CS+_{neu}$	2.14	23	.022[1]	0.44	2.85[1]
	$CS+_{av} > CS-$	1.28	23	.107[1]	0.26	0.78[1]
	$CS+_{neu}$ vs. CS-	-0.72	23	.480[2]	-0.15	0.27[2]
2nd trial block	$CS+_{av} > CS+_{neu}$	1.15	23	.130[1]	0.24	0.67[1]
	$CS+_{av} > CS-$	-0.52	23	.694[1]	-0.11	0.15[1]
	$CS+_{neu}$ vs. CS-	-2.09	23	.048[2]	-0.43	1.34[2]
**Between groups**						
1st trial block	$[CS+_{av} - CS+_{neu}]$	1.80	46	.236[2, B]	0.52	1.06[2]
	$[CS+_{av} - CS-]$	1.30	46	.600[2, B]	0.38	0.57[2]
	$[CS+_{neu} - CS-]$	-0.28	46	1[2, B]	-0.08	0.30[2]
2nd trial block	$[CS+_{av} - CS+_{neu}]$	-0.57	46	1[2, B]	-0.16	0.33[2]
	$[CS+_{av} - CS-]$	-2.50	46	.048[2, B]	-0.72	3.38[2]
	$[CS+_{neu} - CS-]$	-1.97	46	.167[2, B]	-0.57	1.35[2]

t-tests for pairwise comparisons between CS across conditioning groups, within the imagery-based conditioning group, and the classical conditioning group. Tests were conducted one-sided ($CS+_{av} > CS+_{neu}$ and $CS+_{av} > CS-$ for mid-conditioning and post-conditioning ratings) or two-sided (all comparisons for pre-conditioning ratings; all comparisons $CS+_{neu}$ vs. CS-). Positive t-values in the two-sided tests indicate larger values for $CS+_{neu}$. Two-sided t-tests between conditioning groups comparing difference values of CS with Bonferroni-corrected p-values. Positive t-values indicate larger positive differences in the imagery-based conditioning group. Degrees of freedom were corrected for all between-subject t-tests using the Welch method. In this table, uncorrected degrees of freedom are reported. [1]one-sided test, [2]two-sided test, [B]Bonferroni-corrected for three comparisons.

Supplement 6 – Unpleasantness ratings, SCR, and IBI in response to imagery cues

Unpleasantness ratings

Within the imagery-based conditioning group, we analyzed unpleasantness ratings to imagery cues ("How unpleasant do you find the imagery associated with this cue?") before, midway through, and after conditioning. Participants were instructed to indicate unpleasantness on a scale from 0 to 11 or to indicate that they did not associate any imagery with the respective cue. However, as participants rarely made use of the possibility to indicate no imagery (for all 24 participants across three ratings: 2 x $cue_{aversive}$, 3 x $cue_{neutral}$, 5 x $cue_{no\_imagery}$), these cases were recoded to 0.

A Cue Type (aversive vs neutral vs no-imagery) x Time Point (pre vs mid vs post conditioning) ANOVA revealed a significant main effect of Cue Type ($F(2, 46) = 41.52$, $p < .001$, $\eta_p^2 = .802$, $BF_{Incl} = 9.50$ e+13). This effect was followed up by averaging ratings across time points and comparing ratings between cues using *t*-tests. Here, we found significantly higher unpleasantness ratings for aversive vs neutral imagery cues ($t(23) = 9.64$, $p_{one\text{-}tailed} < .001$, $d = 1.97$, $BF_{10,\ one\text{-}tailed} = 1.49$ e+07), for aversive vs no-imagery cues ($t(23) = 10.17$, $p_{one\text{-}tailed} < .001$, $d = 2.08$, $BF_{10,\ one\text{-}tailed} = 3.86$ e+07) , and for neutral vs no-imagery cues ($t(23) = 5.73$, $p_{two\text{-}tailed} < .001$, $d = 1.17$, $BF_{10,\ two\text{-}tailed} = 2{,}748$). There was no significant main effect of Time Point ($F(2, 46) = 1.80$, $p = .176$, $\eta_p^2 = .073$, $BF_{Incl} = 0.39$) and no significant Cue Type x Time Point interaction ($F(4, 92) = 0.61$, $p_{GG} < .579$, $\eta_p^2 = .026$, $BF_{Incl} = 0.09$). The ratings are shown in Figure S8.

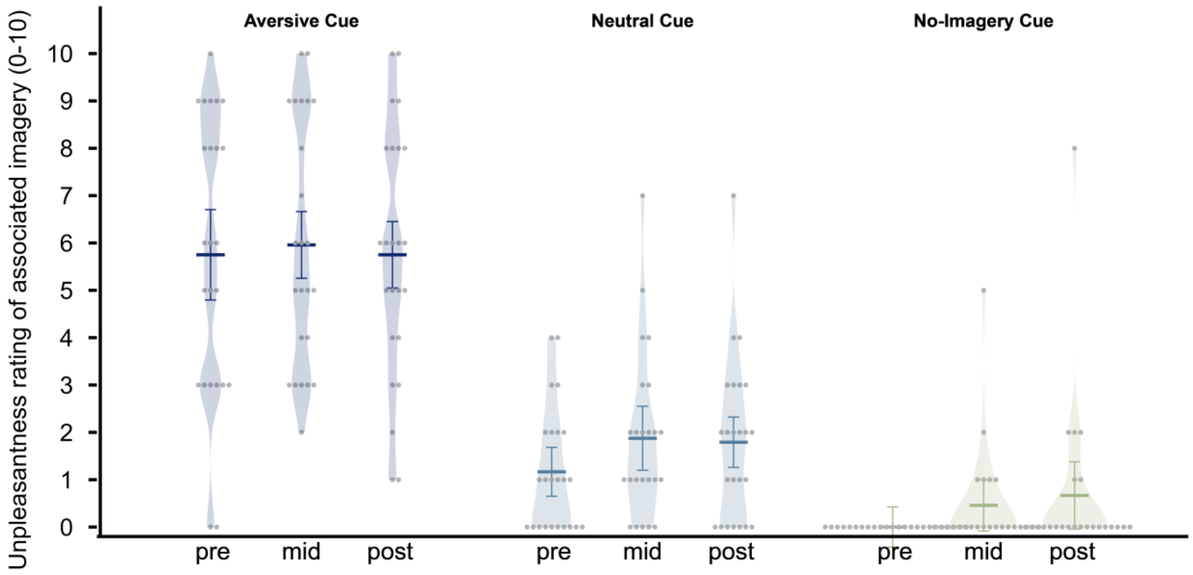

Figure S8. Means and confidence intervals for unpleasantness ratings of imagery associated with cues. *Individual (dots) and mean (horizontal bars) subjective unpleasantness ratings (0 = not unpleasant at all, 10 = extremely unpleasant) at pre-, mid-, and post-conditioning for the imagery associated with each cue. Error bars depict the 95% confidence interval of the mean based on within-subject variance.*

SCR

Cue-evoked SCRs were quantified identically to CS-evoked SCRs, that is, as the normalized average peak from 1 to 5 seconds post-cue after baseline correction. Statistical analyses were identical to CS-evoked SCR amplitudes in the imagery-based conditioning group.

The one-factorial Cue Type (aversive vs neutral vs no-imagery) ANOVA in the imagery-based conditioning group was significant ($F(2, 46) = 3.99$, $p_{GG} = .049$, $\eta_p^2 = .148$, $BF_{10} = 2.79$; Figure S9). This effect was qualified by higher SCR amplitudes to aversive vs neutral imagery cues ($t(23) = 1.89$, $p_{one\text{-}tailed} = .036$, $d = 0.39$, $BF_{10,\ one\text{-}tailed} = 1.88$) and for aversive vs no-imagery cues ($t(23) = 2.18$, $p_{one\text{-}tailed} = .020$, $d = 0.45$, $BF_{10,\ one\text{-}tailed} = 3.06$), replicating previous effects (Mueller et al., 2019). There was no significant difference between neutral and no-imagery cues ($t(23) = 1.05$, $p_{two\text{-}tailed} = .306$, $d = 0.21$, $BF_{10,\ two\text{-}tailed} = 0.35$).

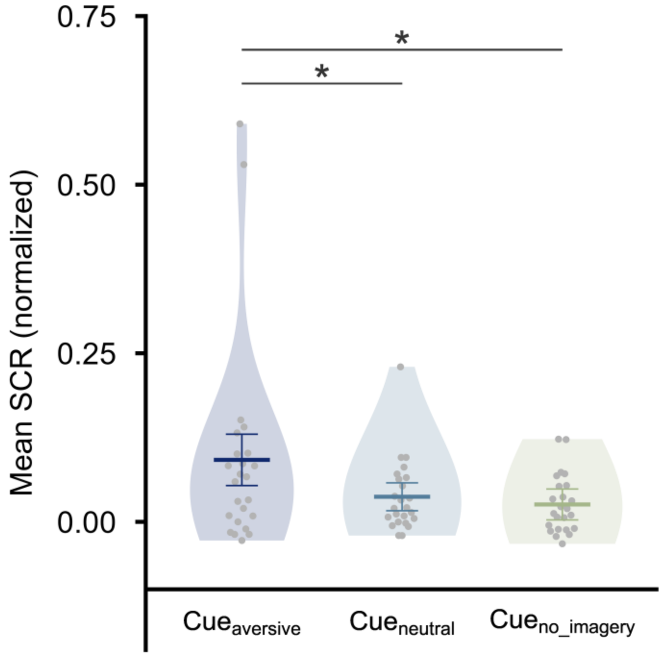

Figure S9. Means and confidence intervals for skin conductance responses to imagery cues. *Individual (dots) and mean (horizontal bars) normalized skin conductance response for each cue in the imagery-based conditioning group. Error bars depict the 95% confidence interval of the mean based on within-subject variance. *p < .05.*

IBI

Cue-evoked IBI was preprocessed identically to CS-evoked IBI. Segments from -1 to 10 seconds relative to the cues were extracted and average IBI from 2 to 5 seconds was used for statistical analyses, consistent with preregistered CS analyses and cue-evoked IBI in previous studies (Mueller et al., 2019). Statistical analyses were identical to CS-evoked IBI in the imagery-based conditioning group with the exception that we expected relative acceleration (i.e. shorter IBI) rather than deceleration for aversive cues compared to the other cues (cf. Mueller et al., 2019).

The one-factorial Cue Type (aversive vs neutral vs no-imagery) ANOVA in the imagery-based conditioning group was significant ($F(2, 46) = 3.79$, $p = .030$ $\eta_p^2 = .141$, $BF_{10} = 2.77$; Figure S10). This effect was qualified by stronger cardiac acceleration to aversive vs neutral imagery cues ($t(23) = -2.00$, $p_{one\text{-}tailed} = .029$, $d = -0.41$, $BF_{10,\ one\text{-}tailed} = 2.25$) and for aversive vs no-imagery cues ($t(23) = -2.74$, $p_{one\text{-}tailed} = .006$, $d = -0.56$, $BF_{10,\ one\text{-}tailed} = 8.45$), replicating previous effects (Mueller et al., 2019). There was no significant difference between neutral and no-imagery cues ($t(23) = -0.55$, $p_{two\text{-}tailed} = .587$, $d = -0.11$, $BF_{10,\ two\text{-}tailed} = 0.25$).

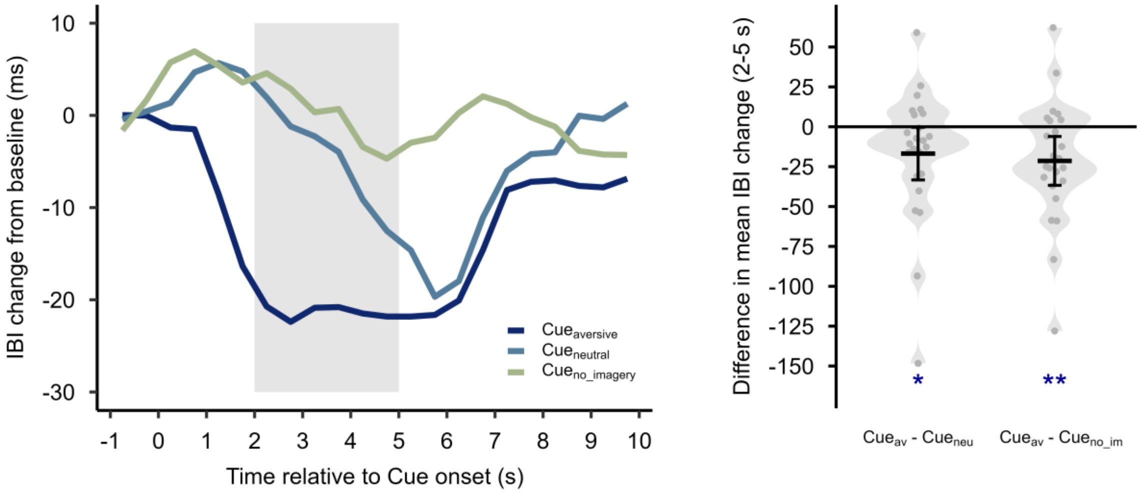

Figure S10. Average IBI to imagery cues. *Interbeat interval (IBI) time course in response to the different cues and differences in mean evoked IBI from 2 to 5 s post-cue (gray area in time course) for the comparisons [Cue_{av} – Cue_{neu}] and [Cue_{av} – Cue_{no_im}]. Individual values are shown as dots, the group means are shown as horizontal bar. Error bars depict the 95% confidence interval of the mean based on within-subject variance. **p < .01, *p < .05.*

Supplement 7 – Frontocentral Theta

Summary

Previous fear conditioning studies (Mueller et al, 2014; Sperl et al., 2019) have shown increased frontocentral theta power to CS+ vs CS-. For the current study, we preregistered supplemental analyses on frontomedial theta.

EEG processing was identical to the LPP pipeline until before segmentation. For theta analyses, EEG was re-referenced against the average and segmented (0 to 2000 ms relative to CS onset). FFT was conducted as implemented in Brain Vision Analyzer, applying a 50% periodic Hamming window on the segmented data first. Theta was operationalized as the mean power density ($\mu V^2/Hz$) from 4 to 8 Hz at Fz.

We conducted ANOVAs identical to the main analyses (CS Type ANOVAs within conditioning groups and a CS Type x Conditioning ANOVA). No main or interactive effects of CS Type on theta power were found in within or across groups (Table S19).

Results

Table S21. ANOVAs on Theta power

	**F**	**df**	**p**	η_p^2	**BF**$_{Incl}$
**Across groups**					
Conditioning Group	0.00	1,46	.976	.000	0.61
CS Type	2.54	2,92	.107	.052	0.56
Group x CS	1.78	2,92	.186	.037	0.44
**Imagery-based**					
CS Type	0.12	2,46	.888	.005	0.13
**Classical**					
CS Type	2.73	2,46	.108	.106	0.87

CS Type x Conditioning Group ANOVA across the two groups. CS Type ANOVAs within the imagery-based conditioning group and within the classical conditioning group. In case of a significant Mauchly test, p-values were corrected using Greenhouse-Geisser correction.

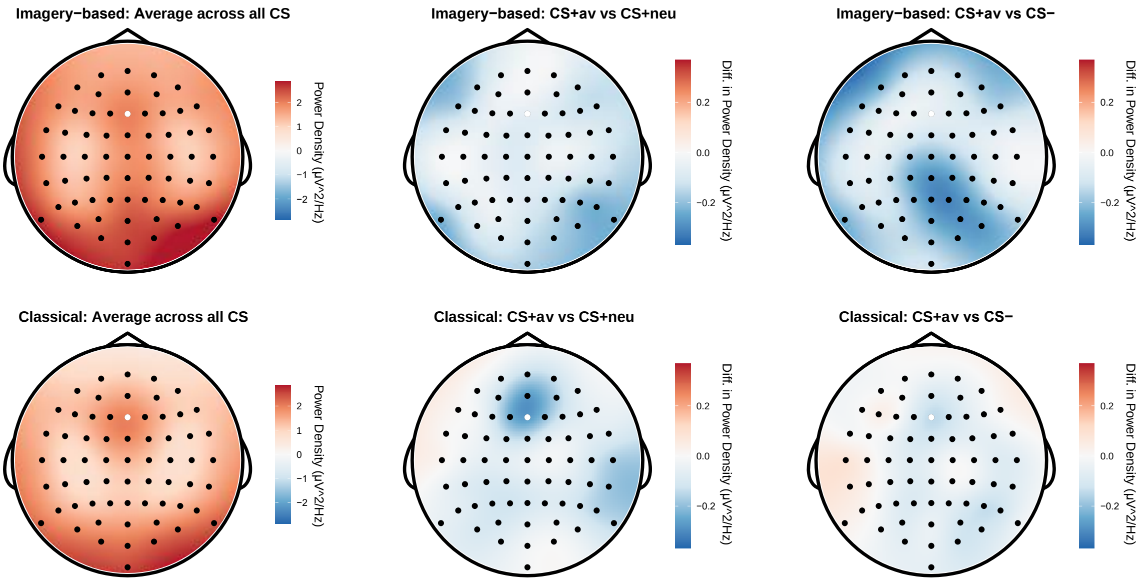

Figure S11. Topographies for theta power. *Topographies depicting theta power (4-8 Hz) averaged across all CS (1st column), as well as difference topographies for CS+$_{av}$ vs. CS+$_{neu}$ (2nd column), and CS+$_{av}$ vs. CS-, separate for the imagery-based conditioning group (1st row) and the classical conditioning group (2nd row).*

References

Mueller, E. M., Panitz, C., Hermann, C., & Pizzagalli, D. A. (2014). Prefrontal oscillations during recall of conditioned and extinguished fear in humans. *Journal of Neuroscience*, 34, 7059-7066. https://doi.org/10.1523/JNEUROSCI.3427-13.2014

Mueller, E. M., Sperl, M. F. J., & Panitz, C. (2019). Aversive imagery causes de novo fear conditioning. *Psychological Science, 30*, 1001–1015. https://doi.org/10.1177/0956797619842261

Rouder, J. N., Morey, R. D., Speckman, P. L., & Province, J. M. (2012). Default Bayes factors for ANOVA designs. *Journal of Mathematical Psychology, 56*, 356-374. https://doi.org/10.1016/j.jmp.2012.08.001

Sperl, M. F., Panitz, C., Rosso, I. M., Dillon, D. G., Kumar, P., Hermann, A., ... & Mueller, E. M. (2019). Fear extinction recall modulates human frontomedial theta and amygdala activity. *Cerebral Cortex*, 29, 701-715. https://doi.org/10.1093/cercor/bhx353

van den Bergh, D., Wagenmakers, E. J., & Aust, F. (2023). Bayesian Repeated-Measures Analysis of Variance: An Updated Methodology Implemented in JASP. *Advances in Methods and Practices in Psychological Science*, *6*, 25152459231168024. https://doi.org/10.1177/25152459231168024
